# Scalable Arrayed T‐cell CRISPR Screening in Autologous Tumor Co‐cultures to Prioritize Combination Partners for T‐cell Engagers

**DOI:** 10.1002/cpz1.70504

**Published:** 2026-10-31

**Authors:** Keenan Ernste, Qiao Zhang, Oscar Cazares, Kai Lu, Servando Palencia, Katerina Hatzi, Celine Eidenshenk, Zhixiang Tong, Yanwen Jiang, Hyun Yong Jin

**Affiliations:** ^1^ Translational Medicine, Genentech South San Francisco CA USA; ^2^ Pathology and Immunology Department Baylor College of Medicine Houston TX USA; ^3^ Translational Genomics and Proteomics Center, Genentech South San Francisco CA USA

**Keywords:** arrayed CRISPR, autologous, bispecific antibody, chronic lymphocytic leukemia, T cell engager

## Abstract

T‐cell‐engaging therapies (TCE) have shown clinical promise in hematologic malignancies, but the T‐cell‐intrinsic determinants of TCE‐mediated cytotoxicity remain incompletely defined. This is in part because functional studies in patient‐derived primary samples have been technically challenging. Here, we describe a flow cytometry‐based arrayed CRISPR knockout screening platform to prioritize regulators of TCE‐mediated cytotoxicity in suspension tumor cells using patient‐derived, autologous chronic lymphocytic leukemia (CLL) samples. The principal advantage of this system is the use of matched T cells and tumor cells from the same patient, retaining patient‐matched effector‐target pairings that are not captured in donor‐mismatched or cell line‐based assays. In this workflow, CD3+ T cells isolated from viably frozen CLL peripheral blood mononuclear cells (PBMC) are expanded and edited for 2 days using arrayed Cas9 ribonucleoprotein nucleofection. TCE‐mediated cytotoxicity was assessed in a 72‐hr, feeder‐supported co‐culture assay using patient‐matched CLL tumor cells in the presence of CD20×CD3 TCE. In the current implementation, the workflow is organized into modules that accommodate up to 96 arrayed perturbations, with technical replicates evaluated under TCE and control conditions. Because perturbations remain physically indexed in an arrayed format, the workflow is amenable to parallelization and laboratory automation, providing a path toward substantially larger targeted screens. This platform therefore provides a scalable framework for mechanistic studies and combination‐target discovery for TCE therapies. © 2026 The Author(s). *Current Protocols* published by Wiley Periodicals LLC.

**Basic Protocol 1**: CD3+ T cell isolation from CLL patient PBMC (**Day 0**)

**Alternate Protocol 1**: Sequential label‐free isolation of CD19+ and CD3+ T cells from healthy donor PBMC

**Alternate Protocol 2**: Sequential label‐free isolation of CD3+ T cells and B‐CLL cells from CLL donor PBMC

**Basic Protocol 2**: Transfer activated T cells to G‐Rex for large‐scale expansion (**Day 4**)

**Basic Protocol 3**: Harvest and CRISPR Editing of Expanded T Cells (**Day 11**)

**Support Protocol 1**: CRISPR knockout optimization and validation in T cells

**Basic Protocol 4**: CLL co‐culture with CRISPR‐edited T cells (**Day 13**)

**Basic Protocol 5**: Flow cytometry analysis of tumor viability and T cell phenotype **(Day 16)**

**Support Protocol 2**: Selecting suitable co‐culture conditions for E:T ratio and TCE concentration for large‐scale screening

## INTRODUCTION

T cell‐engaging therapies (T cell engager, TCE) have emerged as an effective therapeutic modality for hematologic malignancies. However, the determinants of response that regulate T‐cell‐mediated cytotoxicity remain incompletely understood, in part because of technical limitations in interrogating co‐regulators of T‐cell bispecific activity in clinically relevant settings.

Relapsed/refractory chronic lymphocytic leukemia (CLL) remains an area of substantial unmet need despite the clinical benefit achieved with Bruton's Tyrosine Kinase (BTK) inhibitors and venetoclax (Mato et al., 2022). Notably, T cells derived from patients with CLL commonly exhibit multiple features of dysfunction, including senescence‐associated states, increased expression of inhibitory checkpoint molecules, and impaired immunological synapse formation (Goedhart et al., 2023; Ramsay et al., 2008; Riches et al., 2013). Efforts to redirect T‐cell immunity against CLL have had mixed success to date. CD19‐directed CAR‐T cell therapy achieves considerably lower response rates in CLL than in other B‐cell malignancies, an outcome attributed in part to intrinsic T‐cell dysfunction and impaired *in vivo* CAR‐T expansion (Fraietta et al., 2018). Similarly, while CD20×CD3 TCEs such as mosunetuzumab and epcoritamab are under clinical evaluation in CLL, monotherapy activity has been modest, prompting evaluation of combination strategies pairing TCEs with BTK or BCL‐2 inhibition (Marlton et al., 2025; Mhibik et al., 2023). Notably, reducing tumor burden ahead of TCE treatment (e.g., with prior venetoclax) has been associated with improved TCE‐mediated tumor control (Jin et al., 2025), supporting the concept that tumor‐cell burden can directly contribute to impaired T‐cell function in CLL, consistent with earlier observations of CLL cell‐induced defects in immunological synapse formation (Ramsay et al., 2008). Therefore, CLL represents a high‐bar, clinically relevant system for interrogating mechanisms that regulate T‐cell‐mediated cytotoxicity and an especially relevant context for identifying rational combination partners for TCEs aimed at overcoming T‐cell dysfunction and improving anti‐tumor activity.

Conventional discovery approaches are poorly suited to identify causal co‐regulators of TCE cytotoxicity. Correlative analyses derived from patient biomarker data are hypothesis‐generating but non‐causal. Pooled genome‐wide CRISPR screens are generally optimized for capturing survival‐ or proliferation‐based phenotypes in tumors rather than T cell cytotoxicity (Shifrut et al., 2018). Similarly, surrogate readouts such as cytokine production or activation markers do not directly measure tumor elimination. By contrast, our platform, arrayed CRISPR screening in T cells, enables direct functional testing of individual perturbations in edited primary T cells, directly capturing TCE‐mediated cytotoxicity.

An additional advantage of the arrayed architecture is its modularity. Because each perturbation retains a defined physical location, individual gene knockouts can be linked directly to multiple phenotypic readouts and processed as parallel screening units. Arrayed CRISPR approaches have previously been implemented in automated high‐throughput formats, including 384‐well tumor–immune co‐culture assays (Gee et al., 2020). Thus, although the workflow described here was developed for manual execution, its plate‐based organization is compatible with automated liquid handling, parallel cell processing, and automated endpoint acquisition. This provides a potential route to expand the assay from focused screening toward larger hypothesis‐driven gene libraries without sacrificing the direct functional and autologous readouts that distinguish the arrayed approach.

Here, we describe an arrayed CRISPR knockout screening approach using patient‐derived T cells from viably frozen peripheral blood mononuclear cells (PBMCs) together with an autologous CLL co‐culture assay, followed by a step‐by‐step description of the optimized workflow. Figure 1 provides an overview of the workflow and highlights that both effector and target cells are derived from the same patient at the same collection time point. This protocol enables the interrogation of up to 96 arrayed perturbations per nucleofection module in technical triplicate under two treatment conditions (i.e., TCE and CD3‐only binding control) in a single round of assessment, starting from viably frozen CLL PBMCs containing approximately >1 × 10^6^ CD3+ T cells. The use of stromal support in the implementation further enhances the assay's physiological relevance (Hoferkova et al., 2022, 2024). Our screening data show that the platform identified T‐cell‐intrinsic co‐regulators of TCE (Supplementary Fig. 3). By measuring direct tumor cell killing alongside T‐cell expansion, this approach provides a practical framework for studying mechanisms of response to TCE therapy and for identifying targets for combination partners.

**Figure 1:**
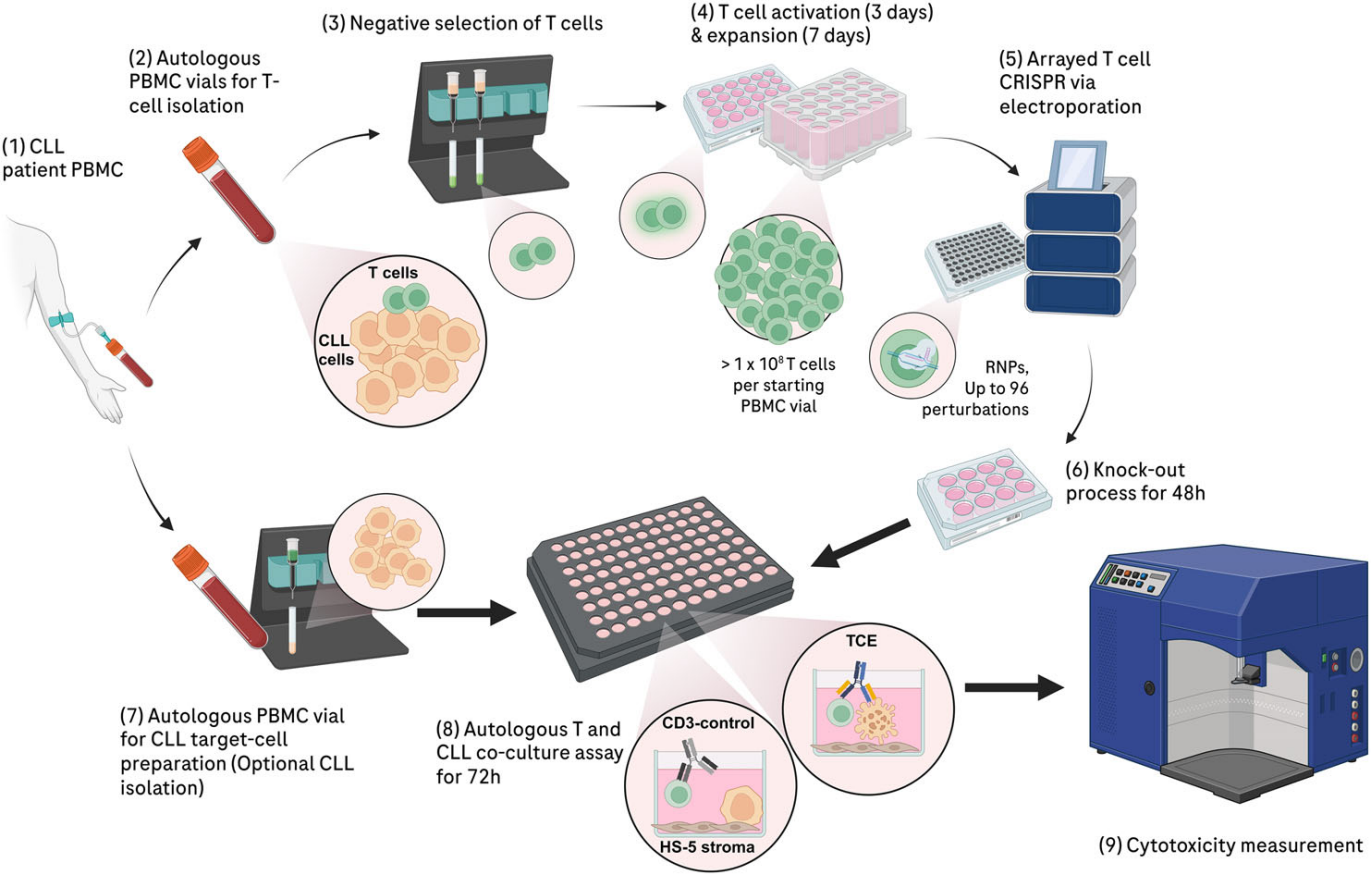
Overview of the autologous arrayed T‐cell CRISPR screening workflow in primary CLL samples. Schematic of the end‐to‐end platform used to identify and prioritize T‐cell‐intrinsic regulators of CD20 × CD3 T‐cell‐engager cytotoxicity in patient‐derived chronic lymphocytic leukemia (CLL). The key feature of the workflow is that both effector T cells and tumor target cells are derived from the same patient with the same blood draw/collection time point, preserving autologous tumor‐immune interactions in a clinically relevant screening format. In a single experimental batch, the platform supports arrayed interrogation of up to 96 arrayed perturbations in technical triplicate. **Step 1**. Viably frozen CLL PBMCs are used as the starting material. **Step 2**. Matched autologous PBMC vials are allocated separately for T‐cell isolation and CLL target‐cell preparation. **Step 3**. CD3+ T cells are enriched by magnetic negative selection. **Step 4**. Isolated T cells are activated and expanded for 10–11 days in large‐scale culture in G‐Rex plates. **Step 5**. Expanded T cells undergo arrayed CRISPR editing using a Cas9 ribonucleoprotein nucleofection in a 96‐well format. **Step 6**. Edited T cells are cultured for an additional 48 hr to allow genome editing and target‐protein loss. **Step 7**. Autologous CLL target cells equivalent to those used in step 1 are prepared from a separate PBMC vial. **Step 8**. Edited effector T cells are added to the matched CLL co‐culture in round‐bottom 96‐well plates and incubated for 72 hr in the presence of either CD20 × CD3 TCE or a CD3‐only binding control antibody. **Step 9**. Endpoint flow cytometry quantifies viable CLL cells and T‐cell phenotypes, with cytotoxicity calculated after normalization to HS‐5 stromal cell spike‐in controls.

### Strategic Planning

Before performing the arrayed CRISPR knockout screen described here, consider the following. The limiting reagent in this autologous screen will generally be the patient‐derived cells used for assay optimization and screening. This experimental approach was designed with high tumor‐burden CLL (≥95% CLL cells) PBMCs obtained from patients with treatment‐resistant disease who had relapsed (Basic Protocol 1). This threshold was chosen so that a PBMC vial can be used directly as the target‐cell source without an additional CLL‐enrichment step (Basic Protocol 4), while remaining pure enough that CD19‐based gating unambiguously identifies tumor cells without meaningful contamination from residual normal B cells (Fig. 7). Samples with lower CLL content can still be used with the enrichment step provided in Alternate Protocol 2. Accordingly, the overall screening workflow described here remains fully applicable to both high‐ and low‐CLL‐purity samples.

**Roadmap**: Basic Protocols 1–5 constitute the main, optimized screening workflow, progressing from T‐cell isolation and activation (Basic Protocol 1), through expansion (Basic Protocol 2) and CRISPR editing with post‐nucleofection recovery (Basic Protocol 3), to autologous co‐culture (Basic Protocol 4) and endpoint flow cytometry (Basic Protocol 5). Figure 1 provides an overview of this workflow, whereas Supplementary Figure 1 guides the selection of the appropriate cell‐preparation strategy based on the starting material. Before undertaking a full screen, Support Protocol 1 should be used to optimize and validate CRISPR editing and target‐protein loss, and Support Protocol 2 should be used to establish suitable effector‐to‐target (E:T) ratios and TCE concentrations. These supporting procedures are preparatory optimization modules rather than additional consecutive steps within each screening run.

There can be significant variability between patients and in disease burden for available PBMC, which may affect assay kinetics. Therefore, it is important to use PBMC with a predetermined tumor burden and cellular subset composition to estimate how many individual CRISPR‐Cas9 knockouts can be performed per sample without running out of biological material.

The Dharmacon™ single guide RNA (sgRNA) pools used in this protocol were purchased from Horizon Discovery. Each gene is targeted by three unique synthetic sgRNAs that are pooled together, with each guide targeting a different region of the gene. Because CRISPR‐Cas9 knockouts rely on DNA repair errors resulting from non‐homologous end joining of double‐stranded breaks, using pooled sgRNAs increases the likelihood of generating genetic damage that results in loss of the target protein. Horizon Discovery's “Cherry Picker” tool enables users to generate custom sgRNA pool libraries in a 96‐well format (https://horizondiscovery.com/en/ordering‐and‐calculation‐tools/cherry‐pick‐library‐loader). We recommend purchasing at least 1 nmol of each sgRNA pool to avoid resuspending the lyophilized RNA in extremely small volumes, which may affect pipetting accuracy and result in suboptimal editing efficiency.

Scalability should also be considered when planning experiments. The CRISPR workflow is naturally organized in modules containing up to 96 gene perturbations. In the current manual implementation, however, the principal limitation is not sgRNA capacity or T‐cell yield but the number of co‐culture plates that can be processed, stained, and acquired efficiently by flow cytometry. The screening experiment shown in Supplementary Figure 3 therefore evaluated approximately 80 genes, corresponding to approximately five 96‐well endpoint plates, with each perturbation assessed in technical triplicate under both TCE and control conditions. Flow cytometry is an important pacing step because prolonged acquisition time can introduce measurable acquisition‐order effects (Fig. 6).

Finally, we optimized these protocols on a 5‐laser Cytek^®^ Aurora spectral flow cytometer. If a comparable spectral flow cytometer is not available, the conditions described here may need to be modified to accurately evaluate the core experimental readouts of tumor cell viability, T cell counts, and pre‐ and post‐editing T cell phenotyping.

*NOTE*: All protocols involving animals must be reviewed and approved by the appropriate Animal Care and Use Committee and must follow regulations for the care and use of laboratory animals. Appropriate informed consent is necessary for obtaining and use of human study material.

## Basic Protocol 1 CD3+ T CELL ISOLATION FROM CLL PATIENT PBMC (DAY 0)

This protocol is optimized for CLL PBMC samples containing ≥95% CLL cells and requires a minimum of two separate PBMC vials for effector and target (CLL) preparation (Supplementary Fig. 1A). In the example workflow, two CLL PBMC vials are used for CD3+ T‐cell isolation and subsequent expansion, and one additional vial is used for preparation of autologous CLL target cells without further purification (see Basic Protocol 4). For instances where the frequency of CLL cells per PBMC vial is lower than 95%, or when users want to optimize T‐cell editing and expansion conditions with healthy donor cells, alternative strategies are provided in separate sections, including isolation of both CD19+ cells and CD3+ T cells from healthy donor PBMCs (Alternate Protocol 1) and sequential label‐free isolation of both CD3+ T cells and the combined pool of B and CLL (B‐CLL) cells from CLL donor PBMCs (Alternate Protocol 2). For a given patient, all PBMC vials used in a screen (for T‐cell isolation and for CLL target‐cell preparation) are cryopreserved aliquots from the same blood draw/collection time point.

### Materials

Viably frozen CLL patient PBMC (Discovery Life Sciences or from NCT05091424 trial (Marlton et al., 2025))
Pan T isolation kit, human (Miltenyi, 130‐096‐535)
Magnetic Activated Cell Sorting (MACS) Buffer (see recipe)
Human IL‐7 Recombinant Protein, PeproTech® (Gibco^,^ 200‐07‐10UG)
Human IL‐15 Recombinant Protein, PeproTech® (Gibco^,^ 200‐15‐10UG)
Anti‐CD3/CD28 beads (Dynabeads™ Human T‐Activator CD3/CD28 Gibco, 11161D)
cOpT medium (see recipe)

LS column (Miltenyi, 130‐042‐401)
QuadroMACS separator (Miltenyi, 130‐091‐051)
Falcon® 24‐well clear flat bottom tissue culture‐treated plate (Corning, 353047)

1 Pre‐warm cOpT T cell recovery medium to 37°C in a water bath.
2 Thaw cryopreserved CLL PBMC rapidly in a 37°C water bath and immediately transfer the entire contents of the vial into 10 ml pre‐warmed cOpT medium. Rest the cells for 5 min in a 37°C water bath before pelleting them by centrifugation at 300–400 × g and room temperature (RT) for 5–10 min. Decant the supernatant into a waste receptacle before resuspending the cells in fresh, pre‐warmed cOpT and determining viable cell count.
  The yield of T cells from a CLL sample often limits this experiment because T cell frequencies are typically very low in patients with high tumor burden. If multiple vials are available, it is advisable to perform a preliminary isolation, flow cytometry phenotyping, and expansion with a single vial to ensure that a sufficient number of T and CLL cells can be obtained before initiating CRISPR editing.
3 For standard specimens, use the Pan‐T cell isolation kit to magnetically sort T cells from the PBMC according to the manufacturer's protocol.
  The protocol was validated using viably cryopreserved CLL PBMC samples, with starting input ranging from 5 × 10^7^ to 1 × 10^8^ total cells and CD3 T cells comprising approximately 1%–3% of the initial cell population.
4 Count the sorted cells and plate them in a 1‐ml volume at 0.5–1 × 10^6^/ml in cOpT medium in a 24‐well tissue culture plate.
5 Add recombinant human IL‐7 (rhIL‐7) and recombinant human IL‐15 (rhIL‐15) to each well at concentrations of 10 ng/ml and 5 ng/ml, respectively. Then, rest the cells overnight in a 37°C incubator.
  Resting the cells overnight is important because the cells need time to recover from the freeze‐thaw process before they can be stimulated. Stimulating the T cells too quickly after thawing may result in significant cell death.
6 After resting the cells overnight (on Day 1), prepare the anti‐CD3/CD28 beads by washing them according to the manufacturer's protocol.
7 Remove the enriched T cells isolated on day 0 from the incubator and count them. Replate the cells in a 1 ml volume at a concentration between 0.5–1 × 10^6^/ml in fresh cOpT medium in a 24‐well tissue culture plate. Add the washed anti‐CD3/CD28 beads to the wells with sorted T cells at a ratio of 3 beads:1 cell, along with rhIL‐7 (10 ng/ml) and rhIL‐15 (5 ng/ml). Gently pipette to thoroughly mix.
  A beads‐to‐cell ratio of 3:1 is higher than the manufacturer's recommended 1:1 ratio. This ratio was chosen based on the CRISPR‐edited T cell expansion protocol previously optimized by (Chang et al., 2024)
8 Incubate T cells with the anti‐CD3/CD28 beads undisturbed for 72 hr in a 37°C incubator.
  CAUTION: During incubation, the T cells should progressively form clumps around the beads as they begin to blast. This process can be observed with a microscope, but do not disturb the blasts by pipetting, or the T cells will expand poorly.

## Alternate Protocol 1 SEQUENTIAL LABEL‐FREE CD19+ and CD3+ T CELL ISOLATION FROM HEALTHY DONOR PBMC

Identifying the appropriate E:T ratio for co‐culture, along with the duration and TCE concentration, is key to successful screening. The goal is to establish conditions that achieve approximately 30% to 40% tumor killing, allowing the screen to capture both enhanced and reduced killing. The assay conditions can be determined with cells isolated from sequential label‐free CD19+ B cells and CD3+ T cells from healthy donor PBMCs. Label‐free isolation was selected to maintain compatibility with the downstream tumor readout, in which live B cells are quantified by CD19‐based gating (Fig. 7). The cell‐preparation protocols based on PBMC source and CLL‐cell content are summarized in Supplementary Figure 1B, contrasting these methods with Basic Protocol 1.

### Materials

Healthy donor PBMCs
REAlease® CD19 MicroBead Kit, human (Miltenyi, 130‐117‐034), the kit includes REAlease Bead Release buffer
REAlease® CD3 MicroBead Kit, human (Miltenyi, 130‐117‐038), the kit includes REAlease Bead Release buffer
Magnetic Activated Cell Sorting (MACS) Buffer (see recipe)
cRPMI cell culture medium (see recipe)

LS column (Miltenyi, 130‐042‐401)
QuadroMACS separator (Miltenyi, 130‐091‐051)

1 Pre‐warm recovery cRPMI medium, equilibrate the MACS buffer to room temperature, and prepare the REAlease Bead release buffer fresh on the day of use.
2 Rapidly thaw cryopreserved healthy donor PBMCs in a 37°C water bath and immediately transfer the contents into 10 ml pre‐warmed cRPMI medium. Rest the cells for 5 min in a 37°C water bath before pelleting at 300–400 × g and RT for 5–10 min. Then, decant the supernatant, resuspend the cells in 5 ml fresh cRPMI, and determine the viable cell count.
3 Pellet the PBMC by centrifugation at 300–400 × g and RT for 5–10 min, and resuspend in 40 µl MACS buffer per 1 × 10^7^ total cells.
4 Add REAlease CD19‐Biotin at 10 µl per 1 × 10^7^ cells, mix, and incubate 5 min at RT.
5 Add REAlease anti‐biotin CD19 microbeads at 50 µl per 1 × 10^7^ cells, mix, and incubate 5 min at RT.
6 Dilute to a maximum of 5 × 10^7^ cells in 500 µl MACS buffer, load onto a pre‐rinsed LS column in a QuadroMACS separator, collect the flow‐through on ice, and wash the column three times with 3 ml MACS buffer.
7 Remove the column from the magnet, add 5 ml freshly prepared REAlease Bead release buffer, flush the retained cells, and incubate the released fraction 10 min (CD19+ cells are now free from MicroBeads and ready for co‐culture assay).
8 Take the CD19‐depleted flow‐through and remove the REAlease complex by pelleting the flow‐through at 300 × g and RT for 10 min,
9 (Optional for sequential T cell isolation) Resuspend in 5 ml MACS buffer and incubate 5 min.
10 (Optional for sequential T cell isolation) Pellet the incubated cells at 300 × g for 5 min, and aspirate the supernatant completely. Resuspend cell pellet in 40 µl REAlease Stop Reagent per 10⁷ total cells and mix well.
11 (Optional for sequential T cell isolation) Proceed with steps 3–8 with CD3‐biotin and anti‐biotin CD3 microbead.
12 Recover the CD3‐positive fractions into cRPMI and allow to rest briefly at 37°C before further manipulation.
13 The isolated B and T cells can be used directly to optimize conditions for the co‐culture assay. To begin optimization of T cell CRISPR editing, follow Steps 6–8 of Basic Protocol 1 to begin the process of T cell expansion, and then proceed to Basic Protocol 2 for large‐scale T‐cell expansion prior to CRISPR.

Healthy donor PBMCs were subjected to sequential label‐free magnetic isolation to enrich first CD19+ B cells and subsequently CD3+ T cells (Table 1). Cell purity and recovery were assessed by flow cytometry. B cells were defined as CD3−CD19+CD20+ and T cells as CD3+CD19‐CD20‐. Both enriched fractions achieved high purity, with ∼95% purity for the CD19+ B‐cell and CD3+ T‐cell fractions. Recovery of CD19+ B cells was high (89.5% of the starting B‐cell population), whereas recovery of CD3+ T cells was more moderate (57.3% of the starting T‐cell population).

**Table 1:** Purity and Recovery After Sequential Label‐free Isolation of CD19+ B cells and CD3+ T Cells from Healthy Donor PBMCs

	**Purity**		**Recovery**	
**Samples**	**Freq. of T cells in total population**	**Freq. of B cells in total population**	**% of starting T cell population**	**% of starting B cell population**
Bulk PBMC (50 × 10^6^ starting cells)	49.3%	6.13%	100%	100%
CD19+ Fraction	0.8%	**93.5%**	0.1%	**89.5%**
CD3+ Fraction	**97.7%**	1.05%	**57.3%**	4.9%

## Alternate Protocol 2 SEQUENTIAL LABEL‐FREE ISOLATION OF CD3+ T CELLS AND B‐CLL CELLS FROM CLL DONOR PBMC

This protocol enables recovery of both autologous T cells and CLL target cells from a single PBMC vial, particularly when the starting sample contains <95% CLL cells and requires tumor‐cell enrichment (Supplementary Fig. 1C). CD3+ T cells are isolated first using releasable magnetic selection, followed by negative‐selection enrichment of CLL cells from the CD3‐depleted flow‐through. This approach provides separately recovered effector and target populations while maintaining compatibility with CD19‐based quantification of viable CLL cells (Fig. 7A and 7B).

When sufficient viable T cells are recovered, the paired populations may be used without prior expansion for small‐scale co‐culture optimization or functional validation using unedited T cells. Basic Protocols 1–3 remain the default T‐cell preparation and editing workflow for large‐scale CRISPR screening; the CLL‐enrichment procedure described here can also be applied to lower‐purity target‐cell samples during co‐culture setup in Basic Protocol 4. In our experience, sequential isolation may yield fewer T cells than the Pan T‐cell isolation method described in Basic Protocol 1, so its suitability should be assessed against the cell numbers required for the intended experiment.

### Materials

Viably frozen CLL patient PBMC (Discovery Life Sciences or from NCT05091424 trial (Marlton et al., 2025))
B‐CLL Cell Isolation Kit (Miltenyi, 130‐103‐466)
REAlease® CD3 MicroBead Kit, human (Miltenyi; 130‐117‐038), including REAlease Bead release buffer
Magnetic Activated Cell Sorting (MACS) Buffer (see recipe)
cRPMI cell culture medium (see recipe)

Falcon® 24‐well Clear Flat Bottom Tissue Culture‐treated plate (Corning, 353047)
LS column (130‐042‐401)
QuadroMACS separator (130‐091‐051)

1 Pre‐warm recovery cRPMI medium, equilibrate the MACS buffer to room temperature, and prepare the REAlease Bead release buffer fresh on the day of use according to the manufacturer's protocol.
2 Rapidly thaw cryopreserved CLL patient PBMC in a 37°C water bath and immediately transfer the contents into 10 ml pre‐warmed cRPMI medium. Rest the cells for 5 min in a 37°C water bath before pelleting at 300–400 × g and RT for 5–10 min. Decant the supernatant, resuspend the cells in 5 ml fresh cRPMI, and determine the viable cell count.
3 Follow the manufacturer's instructions and proceed to T cell enrichment from the PBMC using the REAlease® CD3 MicroBead Kit.
4 After the final step, resuspend the CD3+ fraction in pre‐warmed cRPMI medium at 1–2 × 10^6^ cells/ml and store in a 37°C incubator until analysis (maximum 6 hr).
5 Meanwhile, count the remaining CD3‐ fraction and use it for subsequent B‐CLL cell isolation with a B‐CLL Cell Isolation Kit according to the manufacturer's protocol.
6 After enrichment, resuspend the enriched B‐CLL fraction in pre‐warmed cRPMI medium at 1–2 × 10^6^ cells/ml in an appropriately sized tissue‐culture vessel (i.e., 24‐well tissue culture plate) at 1–2 × 10^6^ cells/ml or other appropriate conditions for downstream application.
  Isolated B‐CLL cells generally have a limited in vitro lifespan that rapidly decreases over the course of a week without subsequent supportive measures (Hoferkova et al., 2022, 2024)^1,2^. Therefore, they should be utilized for further analysis as soon as possible after isolation.
  The required cell number and culture conditions (24‐well) were optimized for the desired cell concentration and expected total cell numbers (1–2 × 10^6^ cells/ml). It can be modified depending on the intended downstream application. For direct co‐culture with freshly isolated T cells or with edited T cells prepared separately, proceed to the co‐culture conditions described in Basic Protocol 4.

## Basic Protocol 2 TRANSFER ACTIVATED T CELLS TO G‐REX FOR LARGE‐SCALE EXPANSION (DAY 4)

The low frequency of T cells in CLL PBMC samples can limit the number of cells available for arrayed CRISPR screening, making *ex vivo* expansion an important preparatory step. Following anti‐CD3/CD28 stimulation in Basic Protocol 1, activated T cells are transferred to G‐Rex plates on day 4 and cultured in cOpT medium supplemented with rhIL‐7 and rhIL‐15 until harvest for CRISPR editing on day 11 (Basic Protocol 3). This protocol covers confirmation of T‐cell blast formation, transfer with minimal disruption of cell aggregates, and replenishment of medium and cytokines to support continued expansion. Figure 2 illustrates expansion kinetics and accompanying changes in CD4/CD8 composition and naïve/memory phenotypes. Supplementary Figure 2 extends this characterization to CD25/FOXP3‐defined populations and additional phenotypic markers within the CD4+ and CD8+ compartments, providing a broader assessment of expansion‐associated changes in the T‐cell populations used for downstream functional assays.

**Figure 2:**
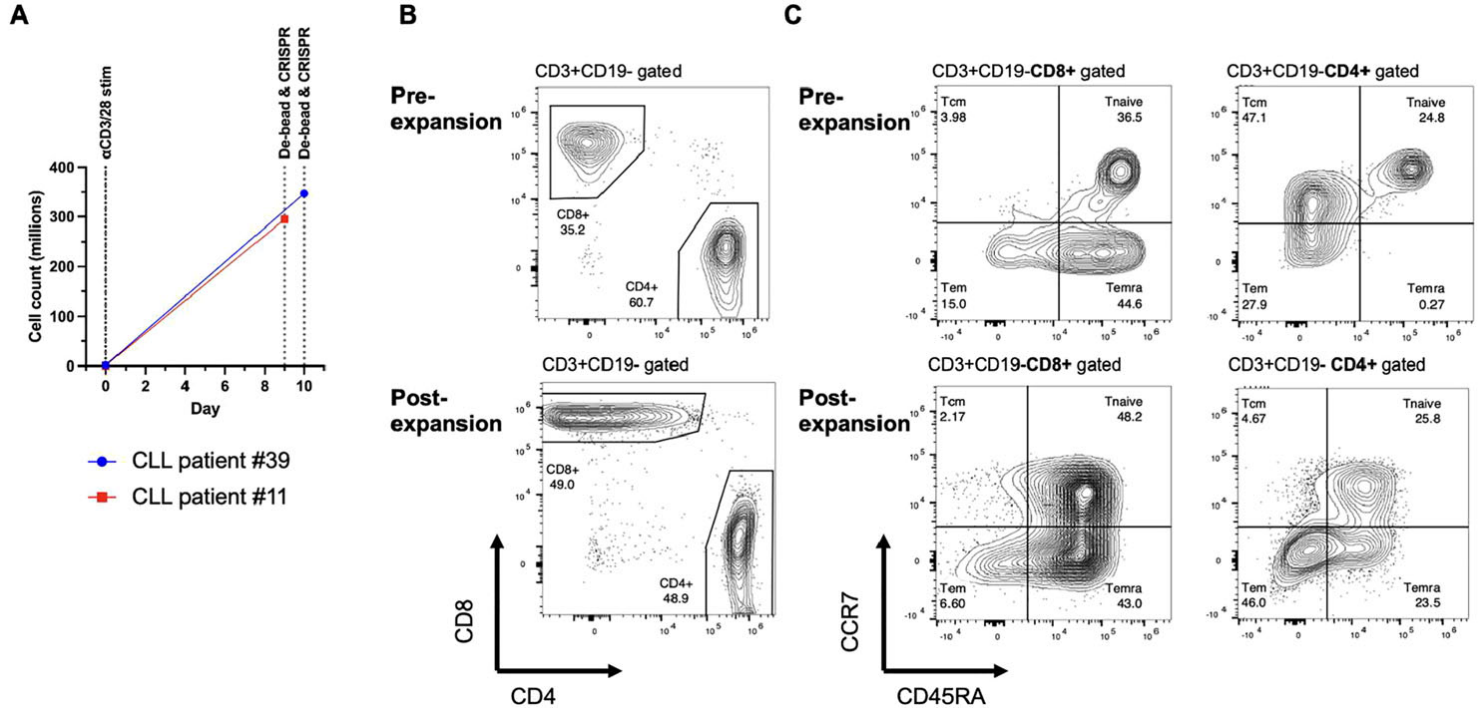
Phenotypic assessment in CLL PBMC‐derived T cells before and after *ex vivo* expansion. Purified CD3+ T cells isolated from PBMCs of a patient with CLL were expanded for 11 days according to **Basic Protocol** **2** **. (A)** Expansion kinetics of purified CLL‐derived T cells during *ex vivo* culture. CD3+ T cells isolated from PBMCs of two patients with CLL were stimulated with anti‐CD3/CD28 beads and expanded under the screening workflow prior to CRISPR editing. Starting from fewer than 1 × 10^6^ purified T cells, both samples expanded by >200‐fold over 9–10 days, yielding sufficient cell numbers for downstream arrayed CRISPR screening. Dashed vertical lines indicate the timing of initial αCD3/CD28 stimulation and subsequent de‐beading/CRISPR editing. **(B)** Representative flow cytometry plots of CD3+CD19‐ gated T cells before and after expansion. Prior to expansion, this donor exhibited a predominance of CD4+ T cells over CD8+ T cells. Following expansion, CD8+ T cells were preferentially enriched, resulting in a more balanced CD4:CD8 composition. **(C)** Differentiation profiles of CD8+ and CD4+ T‐cell subsets before and after expansion, analyzed by CCR7 and CD45RA surface expression. In the CD8+ compartment, expansion increased the relative abundance of naïve‐like T cells and terminally differentiated effector memory T cells re‐expressing CD45RA (Temra), which became the dominant populations. In contrast, the CD4+ compartment showed a marked reduction in central memory T cells (Tcm) and enrichment of effector memory T cells (Tem). Despite these phenotypic shifts, both CD8+ and CD4+ T cells retained substantial naïve‐like cell populations after expansion, comprising 48.2% and 25.8% of the respective subsets. Representative results from two biological replicates were presented. A more detailed surface phenotype assessment post‐expansion is presented in Supplementary Fig. 2. Flow cytometry panel details are presented in Supplementary Table 2A and 2D.

### Materials

cOpT medium (see recipe)
Human IL‐7 Recombinant Protein, PeproTech® (Gibco^,^ 200‐07‐10UG)
Human IL‐15 Recombinant Protein, PeproTech® (Gibco^,^ 200‐15‐10UG)
LIVE/DEAD™ Fixable Blue Dead Cell Stain Kit, for UV excitation (Invitrogen, L23105)
FITC anti‐human CD3 Antibody (Biolegend, 300440)
BD Horizon™ RB705 Mouse Anti‐Human CD4 (BD Biosciences, 570221)
BD Horizon™ BUV737 Mouse Anti‐Human CD8 (BD Biosciences, 612754)
PE anti‐human CD19 Antibody (Biolegend, 302254)
APC/Cyanine7 anti‐human CD45RA Antibody (Biolegend, 304127)
APC anti‐human CD197 (CCR7) Antibody (Biolegend, 353213)

G‐Rex®6 Well Plate (WilsonWolf, 80240M)

1 Following the 72‐hr incubation described in Basic Protocol 1, step 8, examine the cells by light microscopy to confirm the onset of T‐cell blast transformation, as indicated by increased cell size and aggregation/clumping in response to anti‐CD3/CD28 stimulation. If neither T‐cell clumping nor blast transformation is observed, terminate the culture and repeat the procedure beginning with Basic Protocol 1.
2 Gently transfer the entire contents of the wells into the wells of a 6‐well G‐Rex plate. Take care to disturb the clumps of blasting T cells as little as possible during the transfer.
  Combine the cells from up to 2 wells of the 24‐well plate from Basic Protocol 1, step 8, into a single well of the 6‐well G‐Rex® plate. Do not remove the anti‐CD3/CD28 beads from the cells during the transfer.
3 Bring the total volume of medium and cells in each well up to 30 ml with fresh cOpT medium; add rhIL‐7 and rhIL‐15 to a final concentration of 10 ng/ml and 5 ng/ml, respectively. Return the cells to the incubator.
4 On day 9, assess T‐cell expansion with a light microscope. A visible layer of proliferating T cells should be evident, beginning to cover the bottom surface of the wells.
5 Once T‐cell expansion has been confirmed, carefully remove 21 ml medium from the top of each well of the G‐Rex plate without disturbing the cell layer. Replace it with 21 ml fresh cOpT medium. Supplement the culture with rhIL‐7 to a final concentration of 10 ng/ml and rhIL‐15 to a final concentration of 5 ng/ml before returning the plate to the incubator.
6 Culture cells for another two days before proceeding to Basic Protocol 3 for CRISPR editing.

## Basic Protocol 3 HARVEST AND CRISPR EDITING OF EXPANDED T CELLS (DAY 11)

Nucleofection of preassembled Cas9 ribonucleoprotein (RNP) complexes provides a nonviral approach to targeted gene disruption in primary T cells (Oh et al., 2019; Seki & Rutz, 2018). Here, CLL patient‐derived T cells expanded in Basic Protocol 2 are edited in a 96‐well array using pools of three synthetic single guide RNAs (sgRNAs) per gene. This protocol covers T‐cell harvest and removal of activation beads, sgRNA and Cas9 preparation, RNP assembly, nucleofection, and 48 hr post‐nucleofection culture before autologous co‐culture, as described in Basic Protocol 4. Figure 3 and Supplementary Table 1 map the separately prepared T‐cell and RNP inputs to their combination and subsequent nucleofection and recovery steps. Editing efficiency and target‐protein loss are evaluated as described in Support Protocol 1.

**Figure 3:**
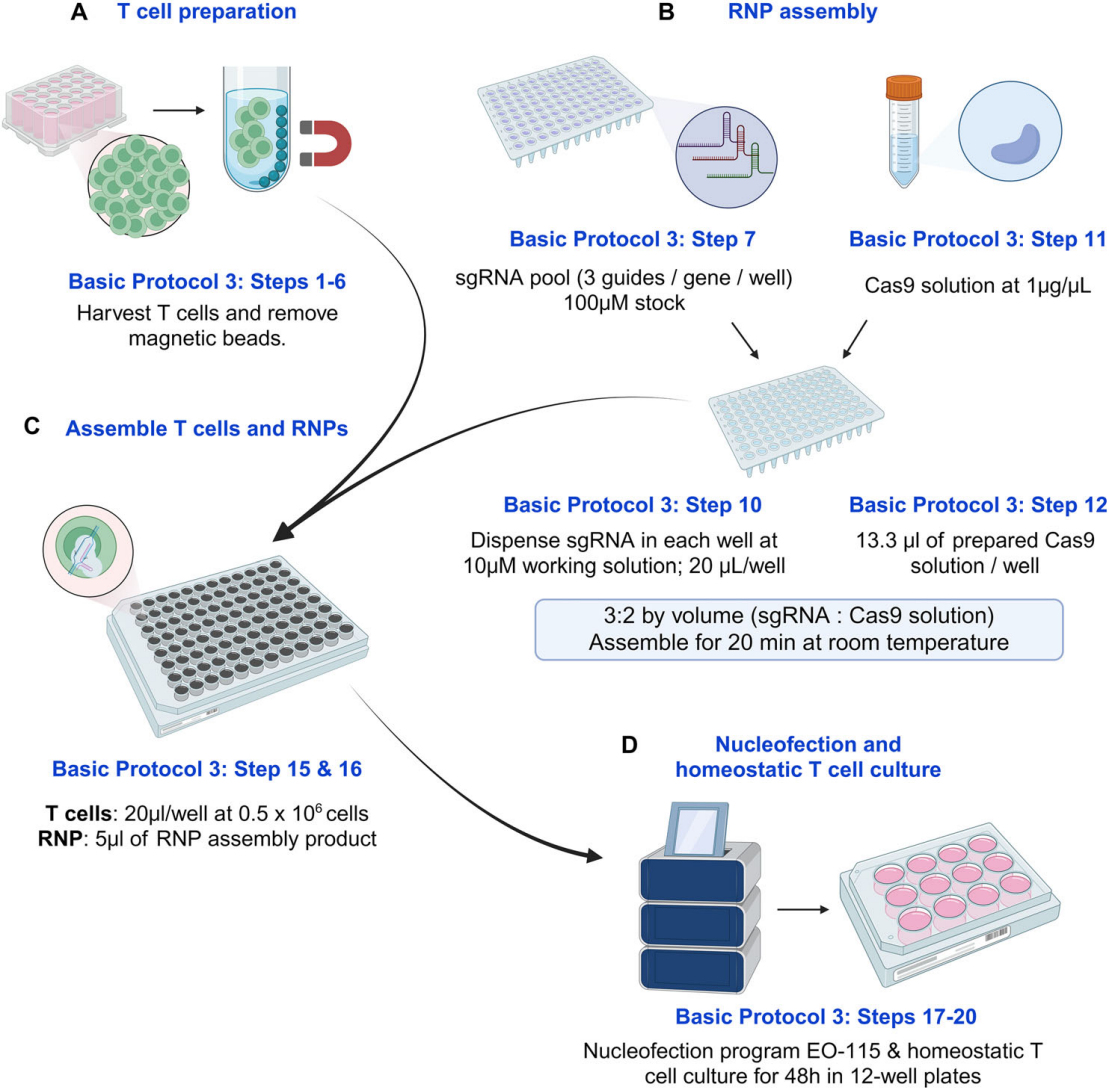
Workflow for Cas9 ribonucleoprotein assembly and arrayed nucleofection of expanded T cells. Schematic of Basic Protocol 3, illustrating the separate preparation of T cells and Cas9 ribonucleoprotein (RNP) complexes, followed by their combination, nucleofection, and post‐nucleofection culture. **(A)** T‐cell preparation. On day 11, T cells expanded according to Basic Protocol 2 are harvested, anti‐CD3/CD28 magnetic beads are removed, and residual aggregates are dispersed. Recovered cells are counted and maintained on ice while editing reagents are prepared (steps 1–6). **(B)** RNP assembly. Pools of three synthetic single guide RNAs (sgRNAs) targeting each gene are reconstituted at 100 µM and diluted to 10 µM working solutions. Each 20 µl sgRNA working solution is combined with 13.3 µl of prepared Cas9 solution (approximately 1 µg/µl), yielding a 3:2 sgRNA:Cas9 ratio by volume. Mixtures are incubated for 20 min at room temperature to assemble RNP complexes (steps 7 and 10–13). **(C)** Combination of T cells and RNPs. Immediately before nucleofection, 0.5 × 10⁶ T cells in 20 µl of P3 buffer containing electroporation enhancer are combined with 5 µl of assembled RNPs in each well of a fresh 96‐well PCR plate (steps 15–16). **(D)** Nucleofection and post‐nucleofection culture. A 21‐µl aliquot from each 25‐µl mixture is transferred to a 96‐well nucleofection plate and nucleofected using program EO‐115. Prewarmed, cytokine‐free cOpT medium is added immediately, followed by a 20‐min recovery at 37°C. Cells are then transferred to 12‐well plates containing cOpT medium supplemented with rhIL‐7 and rhIL‐15 at final concentrations of 10 and 5 ng/ml, respectively, and cultured for 48 hr before autologous co‐culture in Basic Protocol 4 (steps 17–20).

### Materials

Edit‐R predesigned synthetic sgRNA in 96‐well (1 nmol, Horizon Discovery)
Each sgRNA pool contains three sgRNAs targeting the same gene in a single well. This protocol was optimized using Horizon Discovery Dharmacon pooled, synthetic, predesigned sgRNAs, and ≥1 nmol per gene is recommended. Smaller formats can be used, but because this protocol requires resuspension at 100 µM, low‐input preparations require very small volumes, which can reduce pipetting accuracy, particularly in the lyophilized V‐bottom 96‐well plates in which the probes are shipped.
Edit‐R human synthetic AAVS1 sgRNA cutting control (2 nmol, UK‐009310‐01‐02, Horizon Discovery)
Control guide for cytotoxic effects of Cas9‐mediated genome damage without directly affecting gene expression in T cells.
Functional control sgRNA *B2M* (2 nmol, SQ‐004366‐01‐0002, Horizon Discovery)
sgRNA Myc (Horizon Discovery, SG‐003282‐01 (GGTGTGACCGCAACGTAGGA), SG‐003282‐02 (GAGATGGTGACCGAGCTGCT), and SG‐003282‐03 (CGCTGTCTTTGCGCGCAGCC))
sgRNA Lck (Horizon Discovery, SG‐003151‐01 (GTACTACAACGGGCACACGA), SG‐003151‐02 (TCTGGCGCTGTCCACTTAAT), and SG‐003151‐03 (AGCTATGAGCCCTCTCACGA))
Alt‐R Cas9 electroporation enhancer (100nmol, IDT 10007805),
Nuclease‐free duplex buffer (IDT, 11‐05‐01‐03)
Cas9 nuclease (Alt‐R™ S.p. Cas9 Nuclease V3, IDT 1081059)
Nucleofactor Kit (P3 Primary Cell 96‐well Nucleofector® Kit Lonza V4SP‐3096), containing Nucleofaction plate

4D‐Nucleofector® 96‐well Unit (Lonza, AAF‐1003S)
Amaxa 4D‐Nucleofector machine (Lonza)
QuadroMACS separator (Miltenyi, 130‐091‐051)
96‐well PCR plate (ThermoFisher, AB0600)
Falcon® 12‐well clear flat‐bottom tissue culture plate (Corning, 353043)
Fisherbrand™ 25‐ml reagent reservoir (Fisher Scientific, 14‐127‐062)

#### Harvest and prepare T cells for CRISPR editing

1 On day 11, carefully remove the upper 21 ml of medium from each G‐Rex well and retain it in a 50 ml conical tube for subsequent use. Then, using the medium remaining in the G‐Rex well, fully resuspend the T cells and transfer them to a 15‐ml conical tube.
  CAUTION: Before resuspension, inspect the cells microscopically to confirm that they have expanded to near confluence.The bottom surface of the plate is usually almost completely covered with cells.
2 Place the 15‐ml conical tube(s) containing the cells on a magnetic separator and allow the beads to collect along the side of the tube for approximately 30 sec. Transfer the supernatant containing the expanded T cells to a fresh 15‐ml conical tube.
3 Remove the tube containing the beads from the magnetic separator. Add 1 ml of the reserved medium from the 50‐ml conical tube from Step 1 and resuspend the beads thoroughly. Using a 1 ml pipette, pipette vigorously to fully disperse bead aggregates and detach any residual cells bound to the beads.
4 Return the tube to the magnetic separator and allow the beads to recollect for approximately 30 sec. Combine these with the other T cells in the fresh 15‐ml conical tube from step 2.
5 Break up the clumps by gently pipetting and count the recovered T cells to determine cell counts and viability.
  CAUTION: Starting from approximately 1 × 10^6^ T cells from CLL PBMC, expansion typically yields >1 × 10^8^ total cells. Accurate cell counting and efficient CRISPR editing require a uniform single‐cell suspension; if cell clumping persists, centrifuge at 400 × g for 5 min and resuspend the cells 1–2 times as needed to disperse aggregates.
6 Place the cells on ice and immediately proceed with preparation of the CRISPR editing reagents, while prewarming cOpT medium in a 37°C water bath.

#### Prepare the sgRNA pool, Cas9, and post‐CRISPR culture plates before nucleofection

7 Prepare the arrayed sgRNA plate (1 nmol stock, three guides pool per well) by adding 10 µl nuclease‐free duplex buffer, at a final concentration of 100 µM, to each sgRNA well and resuspending the guide material for 30–40 min at room temperature with intermittent gentle pipetting. Spin the plate at 300 × g for a few seconds after resuspension to ensure the sgRNA suspension is evenly distributed across the bottom of the wells.
8 Prepare the post‐nucleofection 12‐well tissue culture plates by labeling each well according to the corresponding gene target, then aliquoting 1 ml fresh cOpT medium containing rhIL‐7 at 11.5 ng/ml and rhIL‐15 at 5.75 ng/ml to each designated well. Pre‐equilibrate the plates in the cell culture incubator at 37°C until ready for use.
  Allocate one well per gene since each gene is subjected to a single nucleofection reaction.
  These concentrations account for the subsequent addition of 150 µl cytokine‐free recovery medium in step 20 and yield final concentrations of 10 ng/ml rhIL‐7 and 5 ng/ml rhIL‐15.
9 Prepare an appropriate amount of P3 buffer that is included in the P3 Primary Cell 96‐well Nucleofector® Kit by first making a complete P3 buffer with the included kit reagents according to the manufacturer's instructions. Then, add Alt‐R Cas9 electroporation enhancer to a final concentration of 4 µM.
  Each well for CRISPR editing will require 0.5 × 10^6^ T cells in a 20‐µl volume (19.2 µl P3 + 0.8 µl Enhancer buffer). Prepare extra to account for pipetting error and place the mixture on ice. For a 96‐well nucleofection, 48 × 10^6^ T cells are required.
  CAUTION: Long‐term exposure to P3 buffer is toxic, so the cells should not be resuspended in P3 buffer until immediately before nucleofection. Carefully read all handling instructions provided by the P3 nucleofection kit manufacturer.
10 Prepare diluted sgRNA working solutions at 10 µM in a 96‐well PCR plate by dispensing 18 µl duplex buffer into each designated well, followed by 2 µl of the corresponding 100 µM sgRNA stock.
11 Prepare the Cas9 protein solution at 1 µg/µl by adding 1.33 µl of Cas9 protein stock (10 µg/µl) plus 12 µl duplex buffer per individual reaction. For experiments involving editing of more than one gene, prepare a Cas9 master mix sufficient for all sgRNA pools to ensure consistency across reactions.

#### Prepare CRISPR‐Cas9 ribonucleoproteins (RNPs)

12 After preparing the nucleofection reagents, combine the diluted 10 µM sgRNA from step 10 with the Cas9 solution from step 11 at a 3:2 volumetric ratio. In the proposed setup, this corresponds to dispensing 13.3 µl of the prepared Cas9 solution into each 20 µl sgRNA pool dilution in the 96‐well PCR plate.
13 Mix well and allow sgRNA and Cas9 to assemble for 20 min at room temperature.
  CAUTION: Although freshly assembled RNPs should ideally be used within 20–30 min of combining Cas9 protein with sgRNAs, preassembled RNPs can be stored for later use. We found that RNPs kept at 4°C remained functional when used the following day, up to 24 hr.
14 While the RNPs are incubating, prepare the nucleofector by turning it on and programming it with the nucleofection plate layout. Use the EO‐115 preset program for nucleofecting activated human T cells.
  We have successfully used both EO‐115 and EH‐115 in different assay settings. The optimal program should be determined empirically based on the specific assay requirements.

#### Assemble T cells and RNPs

15 As the RNP incubation nears completion, centrifuge the T cells prepared in step 6 at 400 × g and RT for 5 min, then resuspend in the P3 buffer/electroporation‐enhancer solution prepared in step 9 at a density of 0.5 × 10^6^ T cells in 20 µl per well. Using a multichannel pipette, dispense 20 µl cell suspension into each well of a fresh 96‐well PCR plate, taking care to avoid introducing air bubbles, which may interfere with nucleofection.
16 As soon as the 20‐min RNP incubation is completed, immediately transfer 5 µl into the wells containing cells in P3/electroporation enhancer solution and mix well.
  The final 5 µl RNP aliquot consists of 3 µl of the 10 µM sgRNA working solution prepared in step 10 (0.3 µl of 100 µM sgRNA + 2.7 µl duplex buffer) and 2 µl of the Cas9 solution prepared in step 11 (0.2 µl of 10 µg/µl Cas9 + 1.8 µl duplex buffer).

#### Nucleofection

17 Use a multichannel pipette to transfer 21 µl volume from each well into the 96‐well nucleofection plate. Avoid introducing air bubbles into the wells. After transferring to all wells, gently but firmly tap the plate on a flat surface several times to distribute the liquid evenly at the bottom of each well. Visually inspect the wells to ensure the solution evenly contacts both metal pieces on the sides of the wells.
  Transfer 21 µl of each prepared 25‐µl solution to the nucleofection plate wells to minimize bubble formation and accommodate pipetting error.
18 Put the included lid on the nucleofector plate, place it in the nucleofector tray, and start the nucleofection using program EO‐115. While the nucleofector is running, prepare a 25‐ml reagent reservoir with the pre‐warmed 37°C cOpT medium with no cytokines added.
19 As soon as the nucleofection completes, immediately add 150 µl of the pre‐warmed cOpT medium to each nucleofected well, and incubate in the cell culture incubator for 20 min for recovery.
  After nucleofection, it is important to quickly add the warmed cOpT medium because cell viability drops rapidly the longer they stay in cold P3 solution.

#### Post‐nucleofection homeostatic culture for T cell resting for 48 hr

20 Transfer 150 µl of the recovered nucleofected‐cell suspension from step 19 into the corresponding pre‐equilibrated 1‐ml culture prepared in step 8. The resulting 1.15‐ml culture contains final rhIL‐7 and rhIL‐15 concentrations of 10 and 5 ng /ml, respectively. Incubate 48 hr before proceeding to Basic Protocol 4.
  A total volume of 171 µl of nucleofected cells was prepared (21 µl nucleofection product + 150 µl cOPT medium from Step 19), of which 150 µl was transferred to the cytokine‐supplemented cOPT medium in 12‐well plates to minimize bubble formation during handling. Because nucleofection was performed with excess cell input, the limited cell loss associated with this transfer did not materially affect downstream assay performance.

## Support Protocol 1 CRISPR KNOCKOUT OPTIMIZATION AND VALIDATION IN T CELLS

Before undertaking large‐scale screening, optimization of CRISPR knockout efficiency is essential. For exploratory studies, healthy‐donor PBMCs may be used, with T‐cell isolation performed using the label‐free isolation procedure described in Alternate Protocol 1, followed by T‐cell expansion according to Basic Protocols 1, 2, and 3. The following protocol describes assessment of CRISPR knockout efficiency in expanded T cells. Arrayed ribonucleoprotein (RNP) editing was benchmarked using AAVS1 as a positive DNA‐damage cutting control with no associated change to the cellular protein pool, and B2M as a phenotypic editing control (Fig. 4). Editing outcomes were evaluated by flow cytometry at 24 hr, 48 hr, and 5 days after editing to define a practical readout window and determine the optimal time point for introducing edited T cells into co‐culture (Fig. 4).

**Figure 4:**
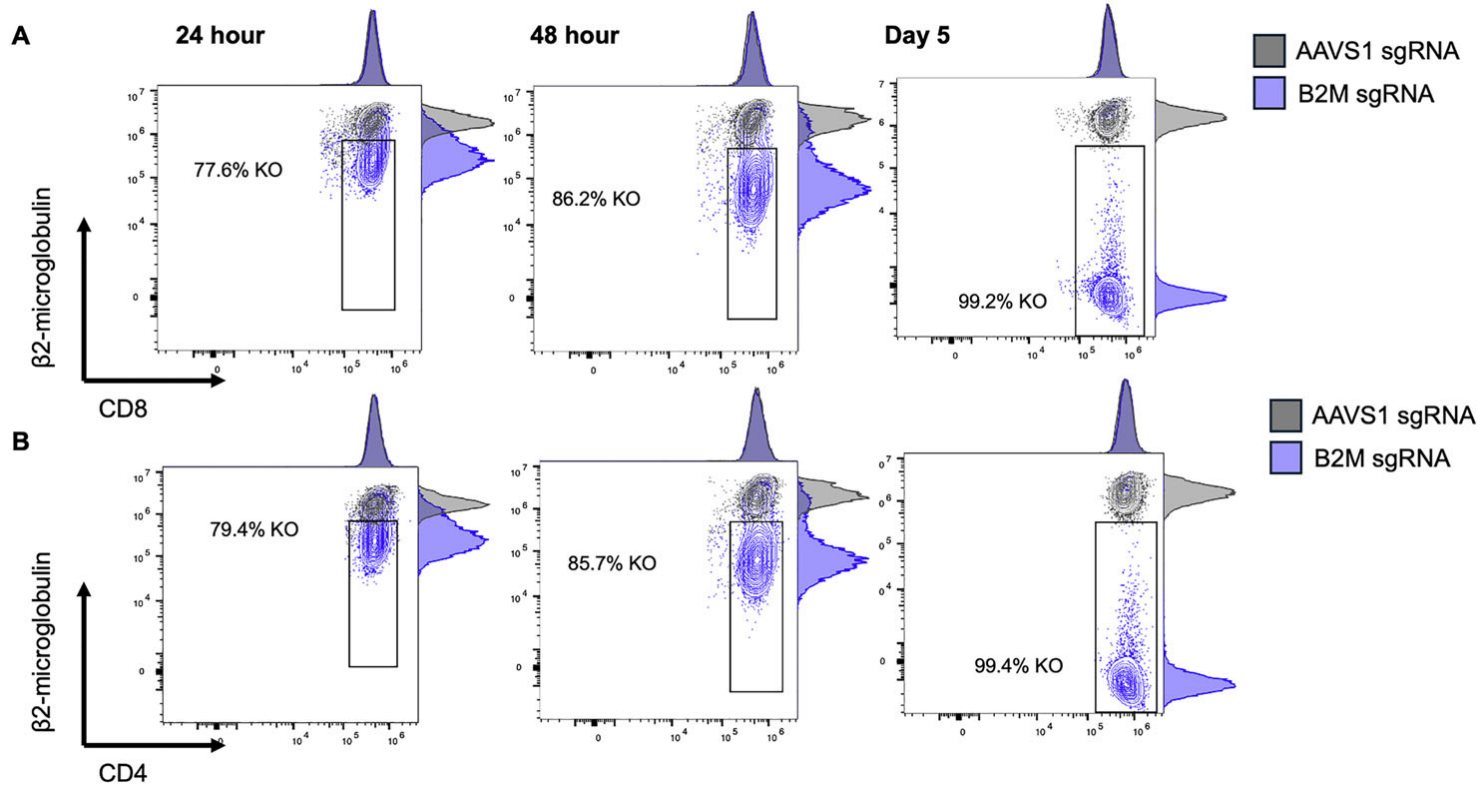
Kinetics of B2M loss after CRISPR editing in T cells from CLL patients. CRISPR knockout was performed in expanded CD3+ T cells derived from CLL PBMCs. Editing kinetics and efficiency were assessed based on surface β2‐microglobulin expression in the CD4+ (A) and CD8+ subsets (B). Editing efficiency was comparable between CD4+ and CD8+ T cells and was not significantly affected by input cell number (data not shown). Representative results are shown for 0.5 × 10^6^ input T cells. Residual β2‐microglobulin expression declined progressively over time, with complete loss observed in most cells by day 5. The 24‐ and 48‐hr data represent the post‐nucleofection incubation period, whereas day 5 reflects target‐loss kinetics over the subsequent 3‐day tumor co‐culture without treatment. *AAVS1*‐sgRNA was used as the guide control. The flow cytometry panel used for detection of T cell β2‐microglobulin knockouts is presented in Supplementary Table 2B.

### Materials

Edit‐R Human Synthetic AAVS1 sgRNA Cutting Control (2 nmol, UK‐009310‐01‐02, Horizon Discovery)
Functional control sgRNA *B2M* (2 nmol, SQ‐004366‐01‐0002, Horizon Discovery)
LIVE/DEAD™ Fixable Blue Dead Cell Stain Kit, for UV excitation (Invitrogen, L23105)
FITC anti‐human CD3 Antibody (Biolegend, 300440, clone UCHT1)
BD Horizon™ RB705 Mouse Anti‐Human CD4 (BD Biosciences, 570221, clone SK3)
BD Horizon™ BUV737 Mouse Anti‐Human CD8 (BD Biosciences, 612754, clone SK1)
PE anti‐human β2‐microglobulin Antibody (Biolegend, 316305, clone 2M2)
FACS buffer (see recipe)

1 Perform CRISPR editing of expanded T cells according to Basic Protocol 3 using *B2M*‐targeting sgRNA and AAVS1‐targeting sgRNA as the phenotypic knockout and editing‐control conditions, respectively.
2 At 24 hr post‐nucleofection, gently resuspend the cultures and collect an aliquot of *B2M*‐ and *AAVS1*‐edited T cells for flow cytometric analysis.
3 At 48 hr post‐nucleofection, collect a second aliquot of *B2M*‐ and *AAVS1*‐edited T cells for flow cytometric analysis. Use the remaining cells for downstream co‐culture according to Basic Protocol 4, if applicable.
4 On day 5 post‐nucleofection, collect the final T‐cell sample to assess target‐protein loss.
  For the experiment shown in Figure 4, day 5 corresponds to 48 hr of post‐nucleofection culture followed by the 72‐hr co‐culture period.
5 Stain each sample with LIVE/DEAD Fixable Blue, CD3, CD4, CD8, and β2‐microglobulin, and acquire cells on a flow cytometer.
6 Gate on viable CD3+ T cells and assess β2‐microglobulin expression separately in the CD4+ and CD8+ T‐cell subsets.
7 Calculate phenotypic knockout efficiency as the frequency of β2‐microglobulin‐negative cells in the *B2M*‐edited condition relative to the corresponding *AAVS1*‐edited control.
8 Compare knockout efficiency and viability across the 24‐hr, 48‐hr, and day‐5 time points to determine whether the standard 48‐hr post‐editing interval provides sufficient target‐protein depletion for the intended downstream assay.

## Basic Protocol 4 CLL CO‐CULTURE WITH CRISPR‐EDITED T CELLS (DAY 13)

The cytotoxic activity of the edited T cells against autologous CLL cells in the presence of a CD20×CD3 bispecific antibody (TCE) can be accurately compared with a 72‐hr co‐culture in a 96‐well format using flow cytometry. The co‐culture design supports *ex vivo* CLL cell survival by adding HS‐5 bone marrow stromal cells. Using a low E:T ratio and saturating concentrations of TCE makes T‐cell cytotoxic potential the limiting factor in tumor cell death, increasing assay sensitivity to perturbation‐dependent differences in T‐cell effector capacity.

### Materials

Viably frozen CLL PBMC
HS‐5 cell line (ATCC CRL‐3611)
cRPMI cell culture medium (see recipe)
  CAUTION: Use round‐bottom 96‐well plates for the co‐culture assay to maintain contact between T cells, HS‐5 cells, and target cells. Plates with wells separated by open space are preferred to minimize liquid transfer between wells. Do not use flat‐bottom plates.
Research‐grade CD20×CD3 TCE (Mosunetuzumab)
CD3‐binding‐only control antibody

25 cm^2^ (T25) cell culture‐treated flask (Corning, 430639)
U‐bottom 96‐well tissue culture plates (VWR, 10062‐902)

#### Prepare CLL donor PBMC cells

1 Start early in the day to ensure the co‐culture proceeds for a full 72 hr by pre‐warming cRPMI medium in a 37°C water bath
  The 72‐hr co‐culture condition was optimized for this protocol (see Support Protocol 2 and Fig. 5). Refer to **Understanding Results** for the rationale underlying this design choice.
  Prior to beginning, calculate the total number of HS‐5, CLL, and T cells necessary to perform each experimental condition at least in triplicate. Culture and handle HS‐5 according to vendor instructions (ATCC CRL‐3611).
  Plan to prepare extra T cells in a separate plate for flow cytometry staining. These will serve as unstained, single‐stained, and isotype‐stained controls to set voltages and gates on the flow cytometer. Leftover AAVS1‐edited T cells can be used for those wells.
  Prepare additional HS‐5 cells for flow cytometry staining controls at the end of the co‐culture.

**Figure 5:**
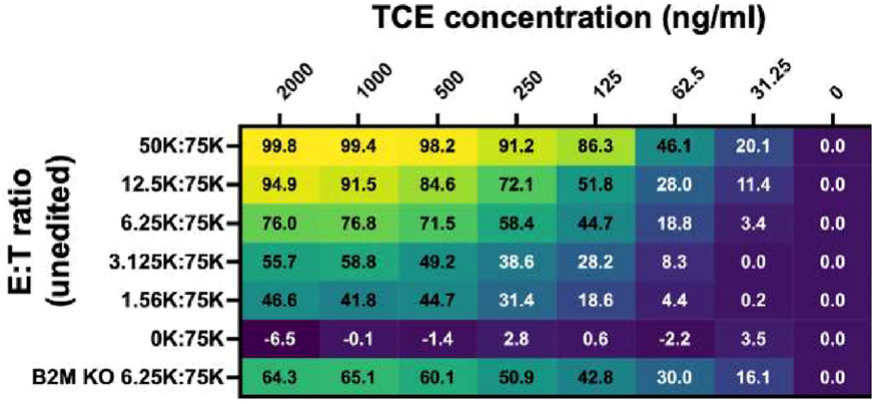
Optimization of screening conditions for TCE‐mediated killing in r/r CLL samples. Relapsed/refractory CLL (r/r CLL) PBMCs were used to define screening conditions for TCE‐mediated cytotoxicity. CD3+ T cells were isolated, expanded for 11 days, and co‐cultured for 72 hr with autologous CLL target cells at varying E:T ratios and TCE concentrations in complete RPMI (cRPMI), as described in **Basic Protocol** **4** and **Support Protocol** **2**. The results demonstrate both dose‐dependent and E:T ratio‐dependent tumor killing. **The *B2M*‐edited benchmark retained TCE‐mediated cytotoxic activity under these conditions**. Based on these studies, the final screening condition was established at E:T = ∼1:50 (1.56K:75K) with 1,000 ng/ml TCE, yielding approximately 40% cell death. This dynamic range allows detection of both enhancement and impairment of TCE‐mediated tumor killing upon individual knockout of genes regulating T‐cell function.

2 Rapidly thaw the required number of autologous CLL donor PBMC vials in a 37°C water bath with gentle swirling.
  Each co‐culture reaction requires 7.5 × 10^4^ CLL cells, so the number of vials needed can be estimated based on a preliminary evaluation of the CLL cell population frequency and the total number of cells per vial.
3 Remove the vial from the water bath when a small sliver of ice remains and transfer it to a biosafety cabinet.
4 Add ∼1 ml pre‐warmed cRPMI dropwise to the vial.
5 Transfer the entire vial contents into 9 ml fresh pre‐warmed cRPMI.
6 Rest the cells for 2–3 min at RT.
7 Centrifuge at 400 × g for 5 min.
8 Decant supernatant and resuspend cell pellet in 10 ml cRPMI.
9 Transfer resuspended cells to a T25 flask and place the flask in the incubator to rest while the remaining co‐culture reagents are prepared

#### Prepare HS‐5 stromal cells

10 Harvest HS‐5 cells at approximately 50% to 80% confluence by trypsinization, then wash and resuspend them in cRPMI.
11 Count cells using an automated cell counter
  Perform at least 5 measurements and calculate the average cell concentration.
  Each co‐culture reaction requires 1.6 × 10^4^ HS‐5 cells per well in 50 µl. Prepare an additional 40 wells for cytometry staining controls.
12 Resuspend the HS‐5 cells at 3.2 × 10^5^ cells/ml in cRPMI.
13 Transfer the suspension to a sterile reservoir and dispense 50 µl into each well of a labeled 96‐well round‐bottom plate using a multichannel pipette, then place the plate into the incubator.
  CAUTION: The accuracy of final assay results relies on accurate and consistent pipetting throughout the co‐culture setup. Use only calibrated multichannel pipettes to transfer cells between wells, and, especially when using many plates, do not allow cells to settle on the bottoms of their respective reservoirs before pipetting them into the co‐culture wells.
  Remix the cell suspension in the reagent reservoir every 60 sec to prevent it from settling to the bottom during pipetting.

#### Prepare TCE and CD3‐binding‐only control antibodies

14 Prepare the antibody dilutions by first thawing the TCE and CD3‐binding‐only control antibodies and keeping them on ice.
  In our experiments, each antibody was diluted to 4 µg/ml in cRPMI, corresponding to 4× the final concentration used in the assay.
  CAUTION: In our optimization experiments, a final concentration of 1 µg/ml was identified as a saturating dose of TCE. However, this should be titrated for each TCE.
15 Place diluted antibodies in a 37°C water bath while preparing the effector T cells.

#### Prepare CRISPR‐edited effector T cells

16 Remove the 48‐hr CRISPR‐edited effector cells in 12‐well culture plates, prepared in Basic Protocol 3, from the incubator.
17 Thoroughly mix the contents using a 1‐ml pipette and transfer 100 µl from each well into 6 wells of a new, labelled 96‐well round‐bottom plate(s) in the same arrangement as the final co‐culture.
  We recommend 3 technical replicates for CD20×CD3 and 3 technical replicates for the CD3‐only binding control antibody for each gene.
  In this setup, 600 µl was prepared for 6 reactions (100 µl in 6 wells) per gene. By arranging the cells in this plate in the same order as the final co‐culture, the cells can be easily transferred into the co‐culture plate(s) using a multichannel pipette after estimating the target effector T cell numbers to be used for the co‐culture at step 21. Also, 100 µl provides sufficient numbers of effector T cells that are optimized in the current protocol (1560 effectors per reaction), but should also cover conditions for higher effector numbers, potentially up to ∼35,000 effectors per reaction)
  The remaining T cells (∼500 µl) can be retained for subsequent assessment of gene‐editing efficiency for each target.
18 Prepare a couple of extra wells containing AAVS1 control‐edited T cells for subsequent cell counting in a separate plate from that prepared in Step 17.
  CAUTION: The well order in which you pipette into the 96‐well plate is recommended to mirror the final well layout for the co‐culture experiment to enable efficient pipetting. Also, collect the remaining CRISPR‐edited effector cells for the wells that will be used as staining controls during subsequent flow cytometry analysis, and for optional sanger sequencing to evaluate knockout efficiency.
19 Once all effector cells have been transferred to the 96‐well round‐bottom plate(s) from Step 17, centrifuge the plates at 400 × g and RT for 3 min to disrupt any cell clumps that may have formed.
20 Without removing the supernatant, resuspend the cell pellet in each well using a multichannel pipette.
21 Calculate how much volume of effector cells to add to the final co‐culture using the extra cells prepared in Step 18, containing AAVS1‐edited effector T cells, using an automated cell counter
  Perform at least 10 counts, calculate the average, and use the resulting cell concentration for all effector wells.
  Some gene knockouts may impact T cell numbers, which will be captured in both tumor‐killing efficacy and T cell numbers measured at the end of screening.
22 Place the effector T‐cell plates in an incubator.

#### Adjust concentrations of CLL donor PBMCs

23 Remove the CLL cells prepared in the T25 flask in Step 9 from the incubator and calculate the total number required to seed 7.5 × 10^4^ CLL cells per well.
  Count the cells using an automated cell counter. Perform 10 counts, calculate the average, and use this value to determine the cell concentration.
  If CLL content is expected to be below 95%, deplete the T cells and enrich for CLL cells according to Alternate Protocol 2.
24 Resuspend the CLL target cells at 1.5 × 10^6^ cells/ml in cRPMI.

#### CLL‐effector co‐culture cytotoxicity assay

25 Now that all co‐culture components have been prepared, begin assembling the co‐culture in the 96‐well round‐bottom plate(s) containing the plated HS‐5 cells by obtaining and organizing sufficient pipette tips and reagents to complete the entire process without stopping.
26 Thoroughly mix the effector cells from step 22 in the incubation plate(s) immediately before transferring to the co‐culture plate.
27 Transfer the desired volume of effector cells from each well of the source 96‐well plate into the corresponding wells of the HS‐5‐containing co‐culture 96‐well plate.
  In our setup, we use 1560 effector cells, which corresponds to 20 to 30 µl of edited T cells.
  1.6 × 10^4^ HS‐5 cells per well in 50 µl per well in step 13.
28 Add 50 µl of either the 4× TCE or 4× CD3‐binding‐only control antibody dilution to the appropriate wells containing effector cells and HS‐5 cells.
29 Finally, add 50 µl of the CLL target‐cell suspension to each well.
  CLL cells prepared in Step 24. With 50 µl per well, 7.5 × 10^4^ CLL cells per well are seeded.
30 Add fresh cRPMI to bring the final volume in each well to 200µl.
  We found that cOpT medium was preferable for T cell expansion (Basic Protocol 2 and Basic Protocol 3), but cRPMI is well validated for use in both T and CLL cultures without affecting T cell killing activity.
31 Gently mix the contents of each well with a pipette and place the plate(s) in an incubator.
32 On the next day and the following day after initiating the co‐culture (protocol days 14 and 15), use a multichannel pipette to gently mix 150 µl in each well by slowly depressing the plunger to the first stop and releasing it three times. Avoid introducing air bubbles or altering the final volume in each well.

## Basic Protocol 5 FLOW CYTOMETRY ANALYSIS OF TUMOR VIABILITY AND T CELL PHENOTYPE (DAY 16)

Flow cytometry can be used to evaluate the viability and phenotypic composition of the co‐culture cells established in Basic Protocol 4. This protocol was optimized to specifically evaluate the total frequency and viability of the CLL and T cells, as well as the surface marker phenotype of the T cells (Figure 7). To account for any differences between wells that are introduced during the staining and acquisition process, this protocol describes how to add antibody pre‐labelled “spike‐in” control HS‐5 cells to each well prior to beginning the flow cytometry staining. By normalizing final population counts to the spike‐in HS‐5 cell counts, population counts can be accurately calculated and compared across CRISPR‐edited conditions to estimate TCE‐dependent tumor cell death and T cell proliferation.

### Materials

FACS buffer (see recipe)
LIVE/DEAD™ fixable blue dead cell stain kit, for UV excitation (Invitrogen, L23105)
APC/Cyanine7 anti‐human CD45 antibody (Biolegend, 304014)
FITC anti‐human CD3 antibody (Biolegend, 300440)
BD Horizon™ RB705 mouse anti‐human CD4 (BD Biosciences, 570221)
BD Horizon™ BUV737 mouse anti‐human CD8 (BD Biosciences, 612754)
PE anti‐human CD19 antibody (Biolegend, 302254)
Spark PLUS UV395™ anti‐human HLA‐DR antibody (Biolegend, 307664)
BD Horizon™ BUV395 mouse anti‐human CD90 (BD Biosciences, 566219)
PE anti‐human β2‐microglobulin antibody (Biolegend, 316305)

#### Flow cytometry reagent preparation

1 Prepare the flow cytometry antibody master mix in FACS buffer at a sufficient volume to dispense 100 µl per well. Store the master mix on ice, protected from light.
2 Prepare the unstained, single‐stained, and isotype controls in a 96‐well round‐bottom plate at a final volume of 110 µl per well.

#### HS‐5 staining as a spike‐in control

3 Trypsinize the HS‐5 cells from the culture plates and resuspend them in 10 ml pre‐warmed cRPMI (See Basic Protocol 4, Step 10 for HS‐5 preparation).
4 Count the HS‐5 cells and resuspend them at 1 × 10^7^ cells/ml in FACS buffer and add 5 µl Spark PLUS UV395 anti‐human HLA‐DR antibody and 5 µl BD Horizon BUV395 mouse anti‐human CD90 antibody per 100 µl cell suspension.
  HS‐5 cells are stained before addition to the experimental samples; therefore, the combined HLA‐DR/CD90 detection in the same spectral region serves as a pre‐label for identification of the spike‐in population rather than as a lineage‐specific phenotypic gate (Fig. 7). The experimental CLL and T‐cell populations are not exposed to these antibodies during this pre‐labeling step and therefore do not acquire the corresponding fluorescence signal.
  Anti‐HLA‐DR alone may provide insufficient staining to identify HS‐5 cells. For optimal population separation and normalization accuracy, use a combination of anti‐HLA‐DR and anti‐CD90 in the same fluorescence channel.
5 Incubate the HS‐5 cells at 4°C for 30 min, protected from light.
6 Wash the cells twice with 5 ml FACS buffer before resuspending in 2 ml FACS buffer and counting with a hemocytometer.
7 Adjust the final HS‐5 cell concentration to 1 × 10^6^ cells/ml in FACS buffer.
8 Once staining begins and throughout the procedure, keep all cells cold by placing plates on ice or at 4°C.

#### Live/Dead and antibody staining for CLL cells

9 Remove the co‐culture plate(s) from the incubator and immediately place on ice.
10 Add 50 µl of the stained HS‐5 cell suspension to each well as a spike‐in control, corresponding to 50,000 HS‐5 cells per well.
11 Centrifuge the plate(s) at 400 × g and RT for 3 min.
12 Decant the supernatant into an appropriate waste container by inverting the plate and flicking the liquid from the wells in one smooth, rapid motion.
13 Wash the cells once with 200 µl PBS.
  CAUTION: Do not use FACS buffer for this wash because it can quench the LIVE/DEAD fixable blue viability dye used in the subsequent set.
14 Decant the supernatant and resuspend the cells in 100 µl LIVE/DEAD stain diluted 1:750 in PBS.
  Prepare the viability dye according to the manufacturer's instructions. For best results, the dilution of LIVE/DEAD Fixable Blue Dead Cell Stain to use should be determined by the user with a separate preliminary titration experiment. Once the dye stock is resuspended in DMSO, it will gradually lose fluorescence over time, even when stored properly at −20°C. Therefore, the dye should be freshly prepared from a lyophilized stock immediately before use.
15 Incubate the cells for 15 min at room temperature, protected from light.
16 Add 100 µl of FACS buffer to each well to quench the residual dye, and centrifuge the plate(s) at 400 × g and RT for 3 min.
17 Decant the supernatant and wash the cells with 200 µl FACS buffer per well.
18 Resuspend the cells in 100 µl of the appropriate antibody master mix prepared in step 1. Mix each well thoroughly by pipetting while adding the staining mix.
19 Incubate the plate(s) at 4°C for 30 min, protected from light.
20 Centrifuge the plate(s) at 400 × g for 3 min.
21 Decant the supernatant and wash the cells twice with 200 µl FACS buffer per well.
22 After the final wash, resuspend the cells in 200 µl FACS buffer.
23 Store the plate(s) at 4°C, protected from light, until acquisition on the flow cytometer on the same day.
  Acquire stained samples as soon as possible after completion of the staining procedure. If immediate acquisition is not feasible, store plates at 4°C, protected from light, and minimize the interval before flow cytometric analysis, as prolonged storage may reduce sample viability and alter apparent cell recovery (Fig. 6).

**Figure 6:**
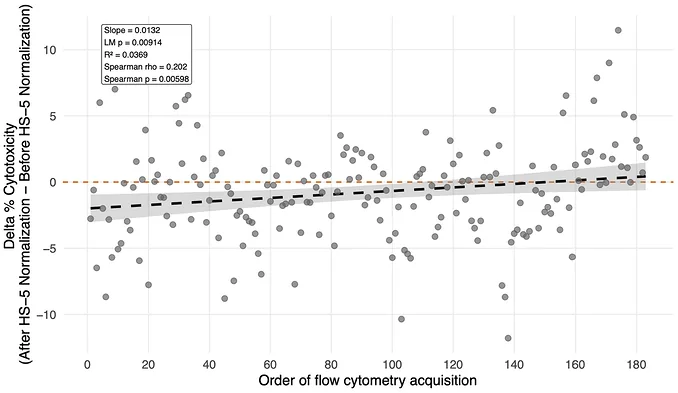
Normalization with HS‐5 spike‐in control corrects acquisition‐order–dependent drift in flow cytometry–based cytotoxicity measurements. A total of 366 co‐culture reactions from CLL PBMC were acquired by flow cytometry over approximately 5 hr, corresponding to 183 paired cytotoxicity measurements (“Order of flow cytometry acquisition”) calculated by comparing TCE‐treated wells with CD3‐binding‐only control antibody‐treated wells. For each pair, % cytotoxicity was calculated before and after normalization to spiked‐in HS‐5 cells, and the difference was plotted as Δ% cytotoxicity (after HS‐5 normalization minus before HS‐5 normalization) versus order of flow cytometry acquisition. Positive Δ% cytotoxicity values indicate that cytotoxicity was underestimated without HS‐5 normalization. Each point represents one paired measurement; the black dashed line indicates the linear regression fit, the gray shading denotes the 95% confidence interval, and the orange dashed line marks no change (Δ = 0). HS‐5 normalization altered calculated cytotoxicity by up to 10 percentage points in either direction and showed a modest but statistically significant positive association with acquisition order (slope = 0.0132 per acquisition rank; linear model p = 0.00914; R² = 0.0369; Spearman ρ = 0.202; p = 0.00598). These data indicate that delayed flow cytometry acquisition can underestimate TCE‐mediated cytotoxicity if HS‐5 normalization is not applied, supporting the inclusion of HS‐5 normalization in the assay workflow.

#### Flow cytometry assessment on viable CLL cells

24 With the flow cytometer, first adjust voltages, acquire the unstained and single‐stained controls, and perform spectral unmixing.
  In Cytek Aurora, it is recommended to manually extract autofluorescence signatures using the AF Explorer tool. Autofluorescence extraction should be performed with an unstained sample. Manually extracting autofluorescence will improve unmixing accuracy because this is a mixed culture, and autofluorescence varies significantly among the HS‐5, CLL, and T cells.
25 Next, acquire isotype controls and gate on the frequencies of pre‐stained HS‐5, viable target CLL cells, and T cells.
26 Once the cytometer settings have been established, remove one co‐culture plate at a time from 4°C storage and acquire the samples promptly to minimize the time cells remain in FACS buffer.
27 Immediately before acquisition, mix the contents of each well thoroughly using a 200 µl pipette.
28 Set the plate shaker to 1300 rpm for 4 sec before acquisition of each well to maintain a uniform cell suspension throughout sample collection.
29 Acquire one of the wells containing a CD3‐binding‐only control antibody at high speed until a minimum of 5000 viable CLL cells are obtained. Record the volume obtained from that well and obtain the same volume from all experimental wells.
  A fixed acquisition volume should therefore be defined from control wells that yields an adequate number of viable CLL events. In our proposed setup, 85 µl from each well was sufficient to obtain 5000 viable CLL cells from the CD3‐only binding control antibody wells. Minimizing the volume needed per well will greatly reduce total acquisition time and the time cells spend in the FACS buffer.
  Extended acquisition periods may lead to artifacts caused by time‐dependent, nonspecific tumor cell death. To ensure robust data interpretation, HS‐5 normalization was applied during analysis to control for these temporal effects.

#### Tumor killing assessment

30 During analysis, identify the viable CLL, viable T‐cell, and HS‐5 spike‐in populations using the predefined gating strategy described in **Understanding Results**, Figure 7A and 7B.

**Figure 7:**
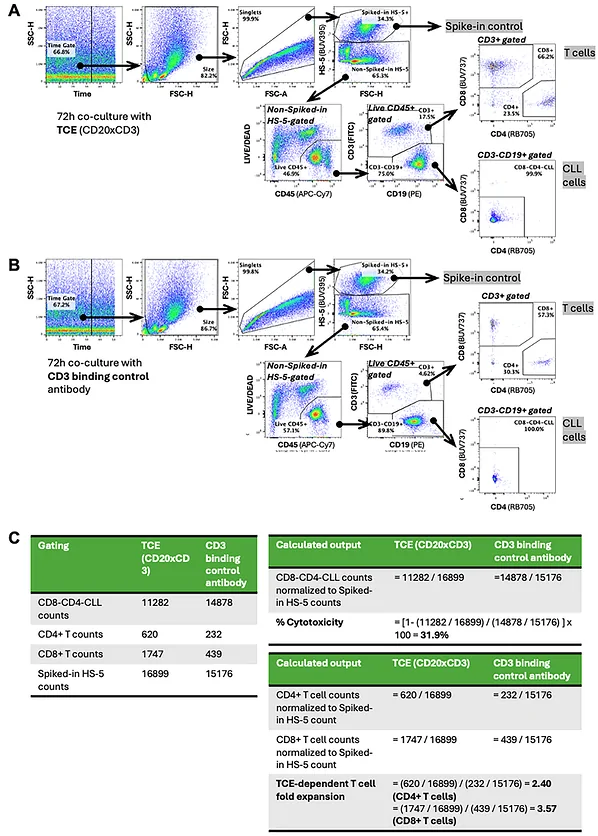
Representative flow cytometry gating strategy and HS‐5–normalized quantification of TCE‐mediated CLL killing and T‐cell expansion. **(A)** Co‐culture treated with 1 µg/ml CD20×CD3 TCE. Sequential gating was performed on acquisition time, cell size, and singlets, followed by identification of pre‐labeled HS‐5 cells used as a spike‐in control for well‐to‐well normalization. The FSC‐A/FSC‐H singlet gate was intentionally permissive to retain the heterogeneous cell populations present in the co‐culture; higher‐FSC events deviating from the main singlet population were subsequently excluded from T‐cell and CLL‐cell quantification through the HS‐5 and live CD45+ gating steps. Within the non‐spiked‐in fraction, viable CD45+ cells were selected to distinguish hematopoietic effector and target cells from unstained HS‐5 feeder cells carried over from the co‐culture. CD45+ cells were then separated into CD3+ T cells and CD3‐CD19+ CLL cells. CD3+ cells were further resolved into CD4+ and CD8+ T‐cell subsets, whereas residual tumor cells were quantified as CD3‐CD19+CD4‐CD8‐ CLL cells. Tumor cells were quantified using CD19‐based gating; CD20 was omitted from the detection panel to avoid potential interference with the CD20‐directed TCE. However, CD20 may be incorporated when clone‐specific non‐interference with the therapeutic antibody has been experimentally confirmed; for the selected CD20 detection antibody, saturating mosunetuzumab did not affect CLL‐cell identification or quantitative CD20 staining (Semerena et al., 2026).  **(B)** Matched co‐cultures were treated with 1 µg/ml CD3‐binding control antibody and analyzed using the same gating strategy as in (A). TCE‐treated and control wells were collected side by side. Detailed flow staining panels are presented Supplementary Table 2C. **(C)** Representative event counts and calculation scheme for assay outputs. CLL cell counts were normalized to spike‐in HS‐5 counts in each well and used to calculate % cytotoxicity in the TCE‐treated well relative to the CD3‐binding control well. Normalized CD4+ and CD8+ T‐cell counts were used to calculate TCE‐dependent fold expansion of each T‐cell subset. In the example shown, TCE treatment induced 31.9% cytotoxicity, together with a 2.40‐fold increase in CD4+ T cells and a 3.57‐fold increase in CD8+ T cells relative to the control condition.

31 Normalize the viable CLL and T‐cell counts in each well to the corresponding HS‐5 spike‐in count to correct for cell loss during staining and for well‐to‐well acquisition variability (See **Understanding Results** and Fig. 7C).
32 Calculate percent cytotoxicity for each condition as:

**Table:** 

**% cytotoxicity** = [1 − (HS‐5 normalized viable CLL count in TCE‐treated wells / HS‐5 normalized viable CLL count in control‐antibody‐treated wells)] × 100
33 Calculate TCE‐induced T cell fold expansion over the CD3‐binding control antibody:

**Table:** 

**TCE‐dependent T cell fold expansion** = HS‐5 normalized viable T cell count in TCE‐treated wells / HS‐5 normalized viable T cell count in CD3‐binding control antibody‐treated wells

## Support Protocol 2 SELECTING SUITABLE CO‐CULTURE CONDITIONS FOR E:T RATIO AND TCE CONCENTRATION FOR LARGE‐SCALE SCREENING

Before large‐scale screening, it is important to optimize the co‐culture conditions and establish a submaximal dynamic assay window, targeting approximately 30% to 40% TCE‐mediated tumor killing (Fig. 5). This range preserves sensitivity for detecting both enhanced and reduced killing phenotypes; the rationale for selecting this assay window and the expected behavior of a well‐performing assay are described in the **Understanding Results** section. Optimization should determine the appropriate E:T ratio and TCE concentration while preserving assay sensitivity to detect both enhanced and reduced killing phenotypes. Initial development of the feeder‐supported autologous CLL co‐culture system was performed in 6‐well plates to evaluate conditions that best maintain CLL viability, including a comparison of CLL cells cultured alone versus on HS‐5 stromal monolayers (Protocol adopted and modified from Hoferkova et al., 2024). The assay was subsequently miniaturized to a 96‐well format for screening and further optimized for target‐cell seeding density, E:T ratio, TCE concentration, culture medium, and assay duration. The assay duration was previously explored, and results from 72 hr co‐culture were presented (Barrett et al., 2023). These optimized conditions should produce measurable but incomplete TCE‐dependent tumor killing while maintaining sufficient dynamic range for bidirectional detection of CRISPR‐associated phenotypes.

### Materials

Viably frozen CLL patient PBMC (Discovery Life Sciences or from NCT05091424 trial (Marlton et al., 2025))
HS‐5 cell line (ATCC CRL‐3611)
cRPMI cell culture medium (see recipe)
25 cm^2^ (T25) cell culture‐treated flask (Corning, 430639)
U‐bottom (round‐bottom) 96‐well tissue culture plates (VWR, 10062‐902)
  CAUTION: Use round‐bottom 96‐well plates for the co‐culture assay to maintain contact between T cells, HS‐5 cells, and target cells. Plates with wells separated by open space are preferred to minimize liquid transfer between wells. Do not use flat‐bottom plates.
Research‐grade CD20×CD3 TCE (Mosunetuzumab)
CD3‐only binding control antibody

1 Define the E:T ratios, TCE concentrations, and co‐culture duration(s) to be evaluated. Use separate plates when comparing different co‐culture durations.
  For the optimization shown in Figure 5, co‐cultures were incubated for 72 hr with 7.5 × 10^4^ CLL target cells per well. Effector‐cell inputs were 5 × 10^4^, 1.25 × 10^4^, 6.25 × 10^3^, 3.125 × 10^3^, 1.56 × 10^3^, or 0 T cells per well. TCE concentrations were 2000, 1000, 500, 250, 125, 62.5, 31.25, and 0 ng/ml. A B2M‐knockout condition at 6.25 × 10^3^ T cells per well was included as an editing benchmark.
2 Thaw the required CLL PBMCs, recover in pre‐warmed cRPMI, centrifuge at 400 × *g* for 5 min, resuspend in fresh cRPMI, and determine the viable cell count. If CLL content is <95%, enrich CLL cells according to Alternate Protocol 2.
3 Harvest HS‐5 cells, determine the viable cell count, and resuspend at 3.2 × 10^5^ cells/ml in cRPMI. Dispense 50 µl per well (1.6 × 10^4^ cells) into a 96‐well round‐bottom cell culture plate.
4 Prepare serial dilutions of the TCE at 4× the desired final concentrations in cRPMI. Prepare the corresponding CD3‐binding control antibody condition.
5 Prepare the effector T cells and determine the viable cell concentration. For CRISPR‐based optimization, use T cells following the 48‐hr post‐nucleofection recovery described in Basic Protocol 3.
  AAVS1‐ or B2M‐edited T cells may be included as benchmark controls; unedited T cells may be used when optimizing co‐culture conditions independently of CRISPR perturbation.
6 Prepare effector T‐cell suspensions corresponding to each E:T condition being tested. Keep the CLL target‐cell input constant while varying the T‐cell input.
7 Adjust the CLL‐cell suspension to 1.5 × 10^6^ cells/ml in cRPMI and dispense 50 µl per well (7.5 × 10^4^ CLL cells).
8 Add the designated effector T‐cell input to each HS‐5‐containing well, followed by 50 µl of the appropriate 4× TCE or control‐antibody solution and 50 µl CLL cells. Add cRPMI to a final volume of 200 µl per well.
9 Incubate the co‐culture at 37°C for the selected duration. For the 72 hr condition, gently mix the wells the next day and the following day.
10 At each selected endpoint (i.e., 72 hr), quantify viable CLL and T‐cell populations by flow cytometry according to Basic Protocol 5 and calculate TCE‐mediated cytotoxicity relative to the matched control condition.

Compare the tested E:T ratios, TCE concentrations, and co‐culture durations, and identify a condition that produces reproducible but incomplete tumor killing.

Use the selected condition for large‐scale screening. In the optimization shown in Figure 5, 1.56 × 10^3^ T cells, 7.5 × 10^4^ CLL cells, 1.6 × 10^4^ HS‐5 cells, 1 µg/ml TCE, and 72‐hr co‐culture produced approximately 40% tumor killing and was selected as the screening condition.

## REAGENTS AND SOLUTIONS

Use sterile ddH_2_O for all preparations unless otherwise stated.

### Complete OpTmizer (cOpT) medium

CTS™ OpTmizer™ T Cell Expansion SFM, bottle format (Gibco, A1048501)
5% Heat‐inactivated Human AB serum (Millipore Sigma, H4522‐100ML)
2 mM GlutaMAX (Gibco, 35050061)
10 mM HEPES (Gibco, 15630080)
100 U/ml Pen‐Strep (Gibco, 15140122)

### Complete RPMI (cRPMI) medium

RPMI 1640 Medium, no glutamine (Gibco, 21870076)
10% Heat‐inactivated Human AB serum (Millipore Sigma, H4522‐100ML)
2 mM GlutaMAX (Gibco, 35050061)
1 mM sodium pyruvate (Gibco, 11360070)
1× MEM NEAA (Gibco, 11140050)
10 mM HEPES (Gibco, 15630080)
50 µM beta‐mercaptoethanol (Gibco, 21985023)
100 U/ml Penicillin‐Streptomycin (Gibco, 15140122)

### Antibodies and Fluorophores

LIVE/DEAD™ Fixable Blue Dead Cell Stain Kit, for UV excitation (Invitrogen, L23105)
APC/Cyanine7 anti‐human CD45 Antibody (Biolegend, 304014, clone HI30, AB_314402)
FITC anti‐human CD3 Antibody (Biolegend, 300440, clone UCHT1, AB_2562046)
BD Horizon™ RB705 Mouse Anti‐Human CD4 (BD Biosciences, 570221, clone SK3, AB_3685591)
PE anti‐human CD19 Antibody (Biolegend, 302254, clone HIB19, AB_2564142)
Brilliant Violet 421™ anti‐human CD20 Antibody (Biolegend, 302330, clone 2H7, AB_10965543)
Spark PLUS UV395™ anti‐human HLA‐DR Antibody (Biolegend, 307664, clone L243, AB_3106239)
BD Horizon™ BUV395 Mouse Anti‐Human CD90 (BD Biosciences, 566219, clone 5E10, AB_2632398)
PE anti‐human β2‐microglobulin Antibody (Biolegend, 316305, clone 2M2, AB_492840)
APC/Cyanine7 anti‐human CD45RA Antibody (Biolegend, 304127, clone HI100, AB_10708419)
APC anti‐human CD197 (CCR7) Antibody (Biolegend, 353213, clone G043H7, AB_10915474)
Brilliant Violet 421™ anti‐human CD366 (Tim‐3) Antibody (Biolegend, 364807, clone A18087E, AB_3068174)
CD279 (PD‐1) Monoclonal Antibody, Brilliant Ultra Violet™ 615, eBioscience™ (Invitrogen, 366‐2799‐41, clone eBioJ105, AB_3093208)
Brilliant Violet 711™ anti‐human CD27 Antibody (Biolegend, 302833, clone O323, AB_11219201)
CD28 Monoclonal Antibody, Brilliant Ultra Violet™ 395, eBioscience™ (Invitrogen, 363‐0289‐41, clone CD28.2, AB_2920946)
PE anti‐human CD57 Antibody (Biolegend, 359611, clone HNK‐1, AB_2562758)
PE/Cyanine7 anti‐human CD25 Antibody (Biolegend, 302612, clone BC96, AB_314282)
PE/Cyanine7 anti‐human CD19 Antibody (Biolegend, 302215, clone HIB19, AB_314245)
PE anti‐human FOXP3 Antibody (Biolegend, 320108, clone 206D, AB_492986)
APC/Cyanine7 Mouse IgG1, κ Isotype Ctrl Antibody (Biolegend, 400127, AB_2892538)

### FACS buffer

Phosphate‐buffered saline, pH 7.4 (PBS; Gibco, 10010023)
2% bovine serum albumin (BSA; Millipore‐Sigma, A9576‐50ML)
0.05% Sodium azide (ThermoFisher Scientific, 014314.09)

### Magnetic Activated Cell Sorting (MACS) buffer

PBS, pH 7.4 (Gibco, 10010023)
0.5% BSA (Millipore‐Sigma, A9576‐50ML)
2 mM EDTA (Invitrogen, AM9260G)

## COMMENTARY

### Background Information

TCEs redirect endogenous T cells toward malignant B cells, but their activity in CLL is shaped by the distinctive dysfunction of the autologous T‐cell compartment. T cells from patients with CLL have been shown to exhibit impaired immunological synapse formation and multiple exhaustion‐ and senescence‐associated features, which can limit effective cytotoxicity (Goedhart et al., 2023; Ramsay et al., 2008; Riches et al., 2013). For this reason, an autologous assay in which effector T cells and tumor cells are derived from the same patient provides a biologically relevant framework for mechanistic studies of TCE response that is not fully captured by donor‐mismatched systems.

A second major consideration is target‐cell biology. Primary CLL cells undergo rapid, spontaneous apoptosis when removed from the patient microenvironment, whereas stromal support can substantially prolong ex vivo survival and better preserve a measurable target population (Lagneaux et al., 1998). Incorporating HS‐5 feeder support improves the interpretability of a 72‐hr co‐culture assay by reducing nonspecific target loss and helping distinguish true TCE‐dependent killing from baseline attrition (Hoferkova et al., 2022). This is especially important in screening mode, where the objective is to detect incremental increases or decreases in cytotoxicity around a partial‐killing baseline.

Arrayed CRISPR is well suited to this application because each perturbation is tested independently, technical replicates can be preserved, and multiple functional endpoints can be measured for the same gene perturbation (Gee et al., 2020). In tumor/immune co‐culture systems, arrayed formats are particularly useful when direct tumor killing must be interpreted alongside other readouts, such as T‐cell accumulation or T‐cell surface phenotype. In the present workflow, parallel measurement of viable CLL cells and viable T cells allows discrimination between genes that primarily regulate T‐cell effector function and those that mainly affect T‐cell growth or fitness (Supplementary Fig. 3).

#### Scalability toward automated and Physical‐AI–enabled screening

Arrayed CRISPR screening is often viewed as a focused complement to pooled genome‐wide screening because its throughput is constrained by physical manipulation of individual perturbations. However, this limitation is principally operational rather than intrinsic to the arrayed format. The present workflow comprises standardized plate‐based operations, including RNP assembly, nucleofection, co‐culture assembly, staining, and endpoint acquisition, that can, in principle, be executed as parallel modules. Laboratory automation has increasingly enabled reproducible scaling of similarly structured biological workflows, and arrayed CRISPR co‐culture has previously been demonstrated in automated 384‐well formats (Gee et al., 2020; Stephenson et al., 2023). In the current manual implementation, approximately 80 gene perturbations were processed in a screening run, with plate handling and endpoint flow cytometric acquisition as the major throughput constraints. Parallel automation could substantially increase the number of modules processed concurrently.

A further extension is the integration of laboratory robotics with artificial‐intelligence (AI)‐based experiment planning, monitoring, and analysis, an emerging paradigm variously described as self‐driving laboratories or Physical AI. Such systems couple computational decision‐making to physical experimental execution and could eventually coordinate parallel plate scheduling, monitor experimental quality, manage acquisition timing, and use accumulating results to prioritize subsequent perturbations or experimental conditions (Tobias & Wahab, 2025). Early biological implementations of agentic‐physical AI have also begun to connect AI‐based reasoning and multimodal monitoring with cell‐culture experimentation, although these approaches remain emerging (Wang et al., 2025). The standardized, modular nature of the current workflow provides a foundation for future automation.

### Critical Parameters

The most important upstream determinant of success is sample quality. Because CLL PBMC samples can contain very low frequencies of recoverable T cells and substantial donor‐to‐donor variability, it is advisable to pre‐screen samples for viability, tumor burden, and CD3+ content before committing material to a full screen. A pilot thaw or small‐scale expansion can prevent the loss of valuable patient material by identifying samples unlikely to support robust expansion or downstream co‐culture.

Efficient editing depends strongly on the activation state and physical quality of the T‐cell culture. Cultures should show clear blast transformation before transfer to G‐Rex and should enter nucleofection as a uniform single‐cell suspension. Fresh Cas9‐RNP assembly and rapid, accurate handling during nucleofection are important because primary T‐cell viability decreases with prolonged manipulation under electroporation conditions (Seki & Rutz, 2018). For new donor samples or new sgRNA pools, inclusion of a cutting control (e.g., *AAVS1*) and a phenotypic control (e.g., *B2M*) is necessary before scaling to a full screen. As we demonstrate, adding functional controls for guide RNA knockout, such as LCK and MYC, which potently regulate TCE‐mediated cytotoxicity, also provides important feedback on screening success.

The co‐culture conditions for screening are also critical. The most informative screening condition is one that yields incomplete killing (approximately 40%) in control‐edited/AAVS1 wells, thereby preserving the dynamic range for both sensitizing and resistance phenotypes. Previous reports with CD20×CD3 bispecific antibodies in primary CLL samples show that cytotoxicity is strongly influenced by E:T ratio, TCE concentration, and treatment duration (Barrett et al., 2023; Mhibik et al., 2023). We therefore optimized the co‐culture assay by systematically evaluating these parameters. Based on this assessment, we selected 1 µg/ml CD20×CD3 TCE with 1.56 × 10^3^ effector cells and 7.5 × 10^4^ target cells per reaction, which achieved approximately 40% specific target‐cell killing after 72 hr of co‐culture.

During flow cytometric readout, acquisition conditions should be standardized across plates. In mixed primary co‐cultures, cells settle quickly, and target viability can decline during prolonged handling. A fixed acquisition volume should therefore be defined from control wells that yield an adequate number of viable CLL events, and plates should be stained and acquired in an order that minimizes time in the FACS buffer before data collection. Normalization to the HS‐5 spike‐in population is particularly valuable for correcting well‐to‐well variation introduced during staining and acquisition.

### Troubleshooting

#### Poor T‐cell recovery or absent blast transformation after activation

This usually reflects low post‐thaw viability, insufficient recovery after thaw, or suboptimal early culture handling. Cultures that fail to form obvious bead‐associated blasts by the time of transfer generally perform poorly in later expansion and editing steps. The most effective corrective actions are to use PBMC samples with good post‐thaw viability and sufficient viable CD3+ T‐cell recovery, preserve the overnight recovery interval before stimulation, and avoid repeated pipetting that disrupts early bead‐associated T‐cell blasts.

#### Low knockout efficiency or excessive cell death after nucleofection

Common causes include weak sgRNAs, incomplete dispersion of cell clumps, delayed use of freshly assembled RNPs, or excessive dwell time in nucleofection buffer. When this occurs, it is advisable to revalidate the guide set in a small‐scale run, confirm that the cells entered nucleofection as a true single‐cell suspension, and move as quickly as possible from P3 resuspension to pulse delivery and warm recovery medium. Primary T‐cell editing studies consistently show that reagent handling and cell state strongly affect editing performance. Editing efficiency also depends on the genome's accessibility to RNP cutting. Genes can be more or less accessible depending on the cell's activation state and the targeted region of the gene. For genes with low knockout efficiency, custom sgRNAs may need to be designed to target more accessible loci. Knockout efficiencies may also need to be evaluated under different conditions of cellular activation and expansion conditions.

#### High well‐to‐well variability at endpoint flow cytometry

High well‐to‐well variability most commonly arises from cell settling during multichannel pipetting, inconsistent washing or decanting, and delayed flow cytometry acquisition after staining. To minimize these effects, cell suspensions in reservoirs should be remixed frequently during plate setup, each well should be resuspended immediately before sample uptake, and plates should be removed from cold storage promptly after staining, with only one plate removed at a time. In our experiments, all samples were collected within 6 hr after staining was completed. Avoiding delayed acquisition is particularly important because prolonged time before analysis can affect apparent tumor cell recovery and lead to progressive underestimation of TCE‐mediated cytotoxicity in samples acquired later in the run. Consistent with this, the data in Figure 6 show that a delay in flow acquisition leads to underestimation of % cytotoxicity without HS‐5 normalization. HS‐5 normalization is therefore recommended as a control for modest acquisition‐related technical variation; however, pronounced outliers or broad well‐to‐well scatter should be interpreted as evidence of sample‐handling error and corrected experimentally rather than computationally. To avoid delays in acquisition, the assay should be standardized to a predefined target volume corresponding to 5000 viable CLL cells from the CD3‐only binding control antibody wells. This should be sufficient to reliably quantify tumor‐cell killing while preserving the minimum required T‐cell representation for accurate analysis.

### Understanding Results

#### Expected assay window for TCE‐mediated cytotoxicity and T‐cell expansion

Under optimized screening conditions, the 72‐hr autologous co‐culture assay is expected to operate within a submaximal cytotoxicity window, with CD20×CD3 TCE treatment producing approximately 30% to 40% tumor killing relative to the matched CD3‐binding control antibody condition (Fig. 5). This response range is desirable for arrayed CRISPR screening because it preserves sufficient dynamic range to detect both sensitizing and resistance phenotypes: baseline killing that is too low reduces sensitivity for identifying perturbations that impair T‐cell function, whereas near‐saturating killing compresses phenotypes that further enhance cytotoxicity. In the CD3‐binding control condition, the endpoint composition of the co‐culture is expected to remain close to the plated E:T ratio of approximately 1:50, consistent with minimal spontaneous T‐cell expansion and limited loss of target CLL cells. In contrast, TCE‐treated wells typically shift to an apparent endpoint ratio approaching ∼1:3, reflecting the combined effects of CLL‐cell depletion and T‐cell expansion over the 72‐hr assay period.

T‐cell numbers are not expected to increase substantially in the CD3‐binding control condition, whereas unedited or AAVS1‐edited control T cells typically show an approximately threefold increase in cell number following TCE treatment. Perturbation of potent negative regulators of T‐cell activation or proliferation can further increase expansion to approximately five‐fold, whereas disruption of genes required for T‐cell fitness, activation, or cell‐cycle progression can markedly reduce or completely abrogate T‐cell expansion (Supplementary Fig. 3). These features are consistent with the behavior expected in arrayed CRISPR screens performed in primary T cells, in which cytotoxicity and proliferative fitness represent related but separable phenotypic outputs. Accordingly, the assay should be interpreted using both residual tumor‐cell abundance and T‐cell accumulation: some perturbations are expected to primarily alter killing capacity, whereas others will predominantly affect T‐cell persistence or expansion, with secondary consequences for tumor clearance.

Although the absolute magnitude of these responses may vary between patient‐derived samples, a well‐performing experiment should reproducibly show three key features: minimal T‐cell expansion in the CD3‐binding control condition, measurable but nonsaturating TCE‐dependent tumor killing, and a clear increase in T‐cell number in the TCE‐treated condition. Together, these anticipated results define a practical operating window for screening and provide a benchmark for distinguishing technical failure from biologically meaningful perturbation.

#### Time‐gated absolute CLL counts as a tumor‐killing readout

In T‐cell‐mediated B‐cell killing assays or tumor‐killing assays that capture hematopoietic cell lineages, cytotoxicity is estimated in multiple ways by flow cytometry. A representative example is cytotoxicity estimated as 1 − (% Live B cells in treatment / % Live B cells in control), where B cells are expressed as a fraction of total CD45+ cells or total inputs (Barrett et al., 2023; Jin et al., 2025; Lu et al., 2026; Mhibik et al., 2023). This approach can be appropriate in co‐culture settings in which non‐B cells are not expected to change substantially during the assay, because shifts in % B cells then mainly reflect loss of target cells. In the present arrayed CRISPR screening format, however, that assumption does not necessarily hold. Because the perturbations are introduced into the effector T cells, knockout of individual genes can substantially alter T‐cell survival and expansion or depletion over the 72‐hr co‐culture period. Under these conditions, % B cells within CD45+ is no longer a specific measure of residual tumor burden; it becomes a composite readout influenced by both CLL‐cell number and T‐cell abundance.

Our assay is designed to directly capture absolute viable CLL‐cell counts derived from the same time gate and normalized to the spiked‐in HS‐5 control population (Fig. 7). Because both the residual CLL events and the HS‐5 spike‐in events are quantified within the same acquisition window, this approach corrects for well‐to‐well variation in recovery and acquisition while avoiding use of the biologically variable CD45+ compartment as the denominator. Operationally, cytotoxicity is calculated as the ratio of viable CLL events to HS‐5 spike‐in events in TCE‐treated wells, relative to matched CD3‐binding control wells, rather than as the fraction of B cells within CD45+ cells. Thus, the tumor‐killing readout is more robust to perturbation‐dependent changes in T‐cell number. A detailed gating strategy and normalization methods are described in Figure 7.

#### Robust detection of benchmark regulators of TCE‐mediated cytotoxicity in an autologous arrayed CRISPR platform

Screening of viably frozen PBMC samples from two independent CLL patients reproducibly detected both decreased and increased TCE‐mediated cytotoxicity, supporting the platform's robustness (Supplementary Fig. 3, left panel). Knockout of proximal TCR signaling genes such as LCK consistently reduced tumor killing, whereas knockout of negative regulators of T cell cytotoxicity reproducibly enhanced killing (Gene names undisclosed). Parallel measurement of fold induction of T‐cell numbers post‐3‐day co‐culture showed that cytotoxicity and expansion can be resolved as distinct assay outputs (Supplementary Fig. 3, right panel): loss of LCK impaired killing with limited effect on T‐cell accumulation, whereas perturbation of MYC markedly reduced T‐cell expansion without impairing tumor killing. Together, these results indicate that the autologous arrayed CRISPR platform is reproducible, functionally discriminating, and suitable for identifying and prioritizing regulators of TCE activity in primary patient‐derived T cells.

#### Protein turnover as a key consideration in interpreting screening results

Interpretation of screening results should be guided by target‐protein depletion, rather than by DNA cutting alone. Although Cas9‐RNP editing occurs rapidly after nucleofection, loss of the encoded protein can lag by several days depending on baseline abundance and protein half‐life. Proteomic studies indicate that protein turnover in T cells is heterogeneous, with some regulatory proteins turning over rapidly whereas more stable housekeeping or structural proteins may persist substantially longer (Wolf et al., 2020). In our workflow, edited T cells are rested for 48 hr before co‐culture, and B2M serves as a practical phenotypic benchmark for target‐loss kinetics. Consistent with its relatively rapid turnover, surface β2‐microglobulin declined progressively by 24 hr and 48 hr and was largely absent by day 5 (Fig. 4), supporting the 48‐hr post‐edit interval as a reasonable starting point for many targets while acknowledging that slower‐turnover proteins may require a longer validation window before co‐culture. Accordingly, top hits emerging from the primary screen should not be interpreted solely based on DNA‐level editing or the screening phenotype itself. Wherever feasible, depletion of the encoded protein should be confirmed independently (for example, by flow cytometry or western blotting), and key findings should be supported by orthogonal follow‐up studies, including pharmacodynamic validation, to strengthen mechanistic interpretation and reduce the risk of overcalling phenotypes due to incomplete or delayed target loss.

#### Interpretation of screening with expanded T cells

*Ex vivo* expansion enables arrayed CRISPR screening but alters T‐cell composition and phenotype (Fig. 2; Supplementary Fig. 2). IL‐7 and IL‐15, together with CD3/CD28 stimulation, can support generation and expansion of memory‐stem‐like T cells from naïve precursors (Cieri et al., 2013), but this does not imply preservation of the original patient‐derived state. Changes in subset frequencies may reflect selective outgrowth or altered marker expression rather than reversal of senescence or exhaustion. Likewise, TIM‐3 can be induced during activation and cytokine‐supported proliferation, and its expression alone does not establish functional exhaustion (Legat et al., 2013; Mujib et al., 2012). Screening hits should therefore be interpreted as regulators of TCE activity in activated, expanded T cells, with prioritized findings validated across independent CLL donors and, where feasible, using minimally manipulated T cells.

### Time Considerations

Once the assay has been optimized, one complete screening cycle requires approximately 16 days from thawed PBMC to endpoint analysis: day 0 T‐cell isolation, day 1 stimulation, day 4 transfer to G‐Rex, day 11 harvest and CRISPR editing, day 13 coculture setup, and day 16 flow cytometric analysis. The most labor‐intensive portions are the initial cell isolation, 96‐well RNP assembly and nucleofection, co‐culture plating, and multicolor staining plus acquisition. In practice, flow cytometry is often the pacing step for larger screening batches, so staining and acquisition should be scheduled to proceed continuously, and plates should not remain in FACS buffer longer than necessary. For a new donor cohort, new sgRNA library, or new TCE molecule, an additional optimization period should be budgeted before the first full screen. For larger implementations, automating plate handling, staining, and acquisition would be expected to reduce this bottleneck and allow multiple 96‐gene screening modules to be processed in parallel.

### Author Contributions

**Keenan Ernste**: Formal analysis; investigation; visualization; writing—original draft; writing—review and editing. **Qiao Zhang**: Investigation; writing—review and editing. **Oscar Cazares**: Investigation; writing—review and editing. **Kai Lu**: Investigation; writing—review and editing. **Servando Palencia**: Investigation; writing—review and editing. **Katerina Hatzi**: Supervision; writing—review and editing. **Celine Eidenshenk**: Supervision; writing—review and editing. **Zhixiang Tong**: Supervision; writing—review and editing. **Yanwen Jiang**: Supervision; writing—review and editing. **Hyun Yong Jin**: Conceptualization; supervision; formal analysis; visualization; writing—original draft; writing—review and editing.

### Conflict of Interest

All authors are employed by Genentech, Inc. Qiao Zhang, Oscar Cazares, Kai Lu, Katerina Hatzi, Celine Eidenshenk, Yanwen Jiang, and Hyun Yong Jin also report shared ownership in Roche.

## Supporting information




*Supplementary Figure 1: Selection of cell‐preparation protocols by PBMC source and CLL cell content*. *Supplementary Figure 2. Extended phenotypic characterization of CLL‐derived T cells before and after ex vivo expansion*. *Supplementary Figure 3: Arrayed CRISPR screening in CLL T cells reproducibly identifies distinct regulators of TCE‐mediated tumor killing and T‐cell expansion from two independent donors Supplementary Table 1. Per‐reaction volumes for sgRNA/Cas9 RNP assembly and nucleofection (Basic Protocol 3)*. Supplementary Table 2A. Pre‐ and post‐expansion T‐cell phenotyping flow cytometry panel used for Figure 2B‐C. *LIVE/DEAD Fixable Blue was used at 1:750 in PBS in a separate viability‐staining step. Supplementary Table 2B. B2M knockout validation panel used for Figure 4. *LIVE/DEAD Fixable Blue was used at 1:750 in PBS in a separate viability‐staining step. *Supplementary Table 2C. Flow cytometry panels used for endpoint co‐culture analysis and T‐cell phenotyping for Figure 7*.

## Data Availability

The data that support the findings of this study are available from the corresponding author upon reasonable request.
