# Supplementary material for: Scalable Arrayed T‐cell CRISPR Screening in Autologous Tumor Co‐cultures to Prioritize Combination Partners for T‐cell Engagers
Source: Curr Protoc. 2026 Oct 31;6(11):e70504. doi: 10.1002/cpz1.70504 (PMC13723204; doi:10.1002/cpz1.70504)
Supplement: Supplementary file 1 [file CPZ1-6-0-s001.docx]

**Supplementary Materials**

**Supplementary Figure 1: Selection of cell-preparation protocols according to PBMC source and CLL-cell content.**

Schematic illustrating the default cell-preparation strategy and two alternative approaches for recovering T cells and B-cell targets. **(A)** Default approach for CLL PBMC samples containing ≥95% CLL cells. Separate patient-matched PBMC vials are allocated for effector- and target-cell preparation. T cells are isolated by Pan T-cell negative selection and activated according to **Basic Protocol 1**, followed by expansion according to **Basic Protocol 2**. A separate PBMC vial serves directly as the source of autologous CLL target cells for **Basic Protocol 4,** without additional CLL-cell isolation. **(B)** Sequential isolation from healthy-donor (HD) PBMCs using **Alternate Protocol 1**. CD19+ B cells are isolated first using a releasable magnetic selection system, followed by isolation of CD3+ T cells from the CD19-depleted flow-through. This approach recovers bead-free B- and T-cell populations from a single vial for assay optimization. The B-cell-first sequence was selected on the basis of the purity and recovery obtained for both populations, as detailed in **Table 1**. **(C)** Sequential isolation from CLL PBMC samples containing <95% CLL cells using Alternate Protocol 2. Bead-free CD3+ T cells are recovered first using releasable magnetic selection, followed by negative-selection enrichment of CLL cells from the CD3-depleted flow-through. The CLL-isolation cocktail targets CD2, CD3, CD4, CD14, CD15, CD16, CD56, CD61, CD235a, FcεRI, and CD34 (Miltenyi: 130-103-466). Because this depletion step also targets T cells and does not incorporate a bead-release step, T cells are isolated beforehand to permit recovery of both populations. CLL cells are recovered as the unlabeled fraction.

**Supplementary Figure 2**. Extended phenotypic characterization of CLL-derived T cells before and after *ex vivo* expansion.

Flow cytometric comparison of T-cell subset composition and marker-positive population frequencies at baseline (black) and after ex vivo expansion (magenta) according to **Basic Protocols 1 and 2**. CD4+ and CD8+ T-cell frequencies are expressed as percentages of CD3+ T cells; the remaining populations are expressed as percentages of the corresponding CD4+ or CD8+ parent population, as indicated on the x-axis. Features assessed include FOXP3+ and CD25+FOXP3− populations within CD4+ T cells; CD366+ and CD279+ populations within each lineage; naïve and memory subsets were defined with CCR7 and CD45RA; and the populations labeled CD28/27−CD57+. Lines connect the corresponding baseline and post-expansion values for each phenotype. Tnaive, naïve/stem-like T cells (CD45RA+CCR7+); Tcm , central memory T cells (CD45RA-CCR7+); Tem, effector memory T cells (CD45RA-CCR7-); Temra, effector memory T cells (CD45RA+CCR7-). Flow panels for the assessment are listed in **Supplementary Table 2D**.

**Supplementary Figure 3: Arrayed CRISPR screening in CLL T cells reproducibly identifies distinct regulators of TCE-mediated tumor killing and T-cell expansion from two independent donors**

**(A) Arrayed T-cell CRISPR screening identifies co-regulators of TCE-mediated tumor killing.** Arrayed CRISPR perturbation of primary T cells in an autologous co-culture assay identified genes that modulate TCE-mediated cytotoxicity. AAVS1-targeting guides were used as a negative control and defined the baseline TCE monotherapy response. Under the screening conditions used, TCE induced approximately 35% tumor killing in the negative-control setting, providing a measurable dynamic range for detection of functional modifiers. The screen identified proximal TCR signaling components, including LCK, as critical positive regulators of TCE-mediated tumor killing. Co-culture condition: 1.56x10^3^ T cells, 7.5x10^4^ CLL cells and 16x10^3^ HS-5 cells per well (96-well plate), 72 hr co-culture with 1 µg/ml TCE in 200 µL culture media. % cytotoxicity was calculated as described in **Basic Protocol 5.** Black dashed line, linear fit; Red dashed line = % Cytotoxicity of AAVS1 (reference gene).

**(B) Arrayed CRISPR screening in primary T cells showed that increased T-cell number can contribute to enhanced tumor killing, although this effect was target dependent.** AAVS1-targeting guides served as the negative control and defined the baseline TCE monotherapy response. Under the assay conditions used, TCE increased T-cell number (CD3+ T cells) by approximately threefold during the 72h co-culture. Notably, the disruption of proximal TCR signaling components, including LCK, impaired tumor killing without materially reducing T-cell proliferation, indicating functional uncoupling of cytotoxicity and expansion. Deletion of MYC abolished T-cell proliferation, confirming the sensitivity of the assay to regulators of T-cell growth.

AAVS1 = functional negative/editing control; B2M = phenotypic knockout-efficiency benchmark; LCK = functional loss-of-killing benchmark; MYC = reduced-expansion benchmark.

| **Stage** | **Step** | **Component** | **Volume (per single reaction)** | **Notes** |
| --- | --- | --- | --- | --- |
| sgRNA resuspension | 7 | Duplex Buffer added to lyophilized 1 nmol sgRNA pool | 10 µL | Yields 100 µM stock; resuspend 30–40 min at RT |
| sgRNA working dilution | 10 | 100 µM sgRNA stock  Duplex Buffer | 2 µL  18 µL | Combine for 10 µM working sgRNA |
| Cas9 dilution | 11 | Cas9 protein stock (10 µg/µL)  Duplex Buffer | 1.33 µL  12 µL | Scale to a master mix for multi-gene batches |
| RNP assembly | 12-13 | Diluted 10 µM sgRNA (from above)  Diluted Cas9 solution (from above) | 20 µL  13.33 µL | Combine at 3:2 (sgRNA:Cas9) volumetric ratio; incubate 20 min at RT |
| Cell/P3/enhancer suspension | 15 | T cells in P3 buffer + 4 µM enhancer | 20 µL  (0.5x10^6^ cells) | Prepared immediately before nucleofection |
| Final RNP addition | 16 | Assembled RNP (3 µL sgRNA fraction + 2 µL Cas9 fraction) | 5 µL | Added directly to the 20 µL cell/P3 suspension |
| Nucleofection transfer | 17 | Combined cell/P3/RNP mix | 21 µL transferred  (of 25 µL total) | Remaining volume minimizes bubble formation during transfer |

**Supplementary Table 1.** Per-reaction volumes for sgRNA/Cas9 RNP assembly and nucleofection (**Basic Protocol 3**).

| **Purpose** | **Marker** | **Fluorophore / channel** | **Working amount per 100 µL** |
| --- | --- | --- | --- |
| Viability | LIVE/DEAD | Fixable Blue | 1:750* |
| T-cell identification | CD3 | FITC | 2.5 µL |
| Exclusion of B/CLL cells | CD19 | PE | 1.25 µL |
| T-cell subset | CD4 | RB705 | 3 µL |
| T-cell subset | CD8 | BUV737 | 3 µL |
| Differentiation phenotype | CCR7 (CD197) | APC | 5 µL |
| Differentiation phenotype | CD45RA | APC/Cy7 | 1.5 µL |

**Supplementary Table 2A**. Pre- and post-expansion T-cell phenotyping flow cytometry panel used for **Figure 2B-C**. *LIVE/DEAD Fixable Blue was used at 1:750 in PBS in a separate viability-staining step. T memory differentiation was defined as follow. Tnaive-like: CCR7+CD45RA+, Tcm: CCR7+CD45RA-, Tem: CCR7-CD45RA- and Temra: CCR7-CD45RA+

| **Purpose** | **Marker** | **Fluorophore / channel** | **Working amount per 100 µL** |
| --- | --- | --- | --- |
|  | LIVE/DEAD | Fixable Blue | 1:750* |
| T-cell identification | CD3 | FITC | 4 µL |
| T-cell subset | CD4 | RB705 | 3 µL |
| T-cell subset | CD8 | BUV737 | 3 µL |
| CRISPR target-protein loss | β2-microglobulin (B2M) | PE | 2 µL |

**Supplementary Table 2B**. B2M knockout validation panel used for **Figure 4.** *LIVE/DEAD Fixable Blue was used at 1:750 in PBS in a separate viability-staining step.

| **Purpose** | **Marker** | **Fluorophore / channel** | **Working amount per 100 µL** |
| --- | --- | --- | --- |
| Viability | LIVE/DEAD | Fixable Blue | 1:750* |
| Hematopoietic-cell identification | CD45 | APC/Cy7 | 1.25 µL |
| T-cell identification | CD3 | FITC | 2.5 µL |
| T-cell subset | CD4 | RB705 | 2 µL |
| T-cell subset | CD8 | BUV737 | 2 µL |
| CLL-cell identification | CD19 | PE | 1.25 µL |
| HS-5 spike-in identification | HLA-DR | Spark PLUS UV395^#^ | 5 µL |
| HS-5 spike-in identification | CD90 | BUV395^#^ | 5 µL |

**Supplementary Table 2C**. Flow cytometry panels used for endpoint co-culture analysis and T-cell phenotyping for **Figure 7.** *LIVE/DEAD Fixable Blue was used at 1:750 in PBS in a separate viability-staining step. ^#^ HLA-DR and CD90 were used together in the same UV395 spectral region to pre-label HS-5 cells before their addition to experimental samples. They therefore serve as an HS-5 spike-in label rather than as lineage-defining markers within the co-culture.

| **Purpose** | **Marker** | **Fluorophore / channel** | **Working amount per 100 µL** |
| --- | --- | --- | --- |
| **Panel 1: T-cell differentiation, inhibitory-receptor and senescence phenotyping (surface)** | | | |
| Viability | LIVE/DEAD | Fixable Blue | 1:750^*^ |
| T-cell identification | CD3 | FITC | 2.5 µL |
| Exclusion of B/CLL cells | CD19 | PE/Cy7^†^ | 1.25 µL |
| T-cell subset | CD4 | RB705 | 3 µL |
| T-cell subset | CD8 | BUV737 | 3 µL |
| Differentiation phenotype | CCR7 (CD197) | APC | 5 µL |
| Differentiation phenotype | CD45RA | APC/Cy7 | 1.5 µL |
| Inhibitory receptor | CD366 (TIM-3) | BV421 | 4 µL |
| Inhibitory receptor | CD279 (PD-1) | BUV615 | 4 µL |
| Co-stimulatory receptor / senescence | CD27 | BV711 | 3 µL |
| Co-stimulatory receptor / senescence | CD28 | BUV395 | 3 µL |
| Senescence | CD57 | PE | 3 µL |
| **Panel 2: CD4+ regulatory T-cell phenotyping (surface + intracellular)** | | | |
| Viability | LIVE/DEAD | Fixable Blue | 1:750^*^ |
| T-cell identification | CD3 | FITC | 2.5 µL |
| T-cell subset | CD4 | RB705 | 3 µL |
| Activation / Treg phenotype | CD25 | PE/Cy7 | 4 µL |
| Treg phenotype (intracellular) | FOXP3 | PE | 4 µL^‡^ |

**Supplementary Table 2D**. Extended pre- and post-expansion T-cell phenotyping flow cytometry panels used for Supplementary Figure 2. Panels 1 and 2 were stained in separate wells using the same pre- and post-expansion T-cell samples. ^*^LIVE/DEAD Fixable Blue was used at 1:750 in PBS in a separate viability-staining step. ^†^CD19 was used to exclude residual B/CLL cells; the PE channel in Panel 1 was assigned to CD57. ^‡^FOXP3 was stained intracellularly after surface staining (CD3, CD4, CD25) and fixation/permeabilization with the Foxp3/Transcription Factor Staining Buffer Set according to the manufacturer’s instructions. Gates were set using fluorescence-minus-one (FMO) controls for LIVE/DEAD, CD279, CD45RA, CD27, CD28, CD25, and FOXP3, and isotype controls for CD3, CD19, CD366, CCR7, CD57, and FOXP3. T-cell differentiation subsets were defined as follows. Tnaive-like: CCR7+CD45RA+, Tcm: CCR7+CD45RA−, Tem: CCR7−CD45RA−, and Temra: CCR7−CD45RA+.
